# Interplay and cooperation of *Helicobacter pylori* and gut microbiota in gastric carcinogenesis

**DOI:** 10.1186/s12866-021-02315-x

**Published:** 2021-09-23

**Authors:** Seyedeh Zahra Bakhti, Saeid Latifi-Navid

**Affiliations:** Department of Biology, Faculty of Sciences, University of Mohaghegh Ardabili, 56199-11367 Ardabil, Iran

**Keywords:** *H. pylori*, gut microbiota, interaction, gastric carcinogenesis

## Abstract

Chronic *Helicobacter pylori* infection is a critical risk factor for gastric cancer (GC). However, only 1–3 % of people with *H. pylori* develop GC. In gastric carcinogenesis, non-*H. pylori* bacteria in the stomach might interact with *H. pylori*. Bacterial dysbiosis in the stomach can strengthen gastric neoplasia development via generating tumor-promoting metabolites, DNA damaging, suppressing antitumor immunity, and activating oncogenic signaling pathways. Other bacterial species may generate short-chain fatty acids like butyrate that may inhibit carcinogenesis and inflammation in the human stomach. The present article aimed at providing a comprehensive overview of the effects of gut microbiota and *H. pylori* on the development of GC. Next, the potential mechanisms of intestinal microbiota were discussed in gastric carcinogenesis. We also disserted the complicated interactions between *H. pylori*, intestinal microbiota, and host in gastric carcinogenesis, thus helping us to design new strategies for preventing, diagnosing, and treating GC.

## Background

*Helicobacter pylori* infection is the critical risk factor for gastric cancer (GC) [1–4]. Inflammation and injury induced by *H. pylori* can continuously damage the function and architecture of the gastric epithelium [5]. However, it should be mentioned that the successful removal of *H. pylori* does not necessarily inhibit the GC development [6]. Thus, there may be other factors involved in the carcinogenesis of GC which require further research. Numerous intestinal and gastric microbes have been known as procarcinogens in colorectal cancer and GC [7–11], or probiotics that increase patients’ immunotherapy response with cancer [12]. However, there are few reports about microbiota composition in precancerous lesions.

Normal intestinal flora (IF) has been indicated to accelerate the beginning of gastrointestinal intraepithelial neoplasia (GIN) and increase its development [13]. Non-*H. pylori* bacteria, pathogenic or commensal IF, may colonize the stomach and show the excessive risk of gastric adenocarcinoma, especially in susceptible patients with *H. pylori* [14, 15]. INS-GAS (insulin-gastrin) transgenic mice with high levels of circulating gastrin develop spontaneous atrophic gastritis and GIN with an 80 % prevalence 6 months after *H. pylori* infection. Evaluation of this model revealed that commensal intestinal bacteria may promote GC. [16, 17]. Male restricted ASF (a restricted microbiota confined to three species of Altered Schaedler’s Flora) and IF INS-GAS mice presented gastric pathology as the Correa model, even without *H. pylori* infection [18]. Although ASFs are beneficial for mice [19], it appears that their colonization in the stomach may be involved in the production of various oxidizing agents, oxygen radicals, nitrosamines, and genotoxic compounds and mutagens. Various studies have shown that human gastric colonization with bacteria other than *H. pylori* such as *Actinobacteria*, *Proteobacteria*, *Fusobacteria*, *Firmicutes*, and *Bacteroides* (many are colonized normally in the lower intestine) can affect the gastric adenocarcinoma risk [17, 20–22]. *Lactobacillus* is a facultative anaerobe representing a gut microbiota component and is in general a probiotic in-transit passenger. The gastric mucosa colonization by *Lactobacillus* shows an alteration in cancerous patients with gastric microenvironment. A study in Taiwan showed that *Lactobacillus* is abundantly found in GC patients. This seems to be due to the use of probiotic microbes as a dietary supplement [23].

Gastritis can change the fecal microbiome composition, which might possibly be aggravated by *H. pylori* infection [24]. The gut microbiome changes could be associated with chronic gastrointestinal diseases, the close interaction of *H. pylori* infection, gut microbiome, and gastritis [24]. Numerous pieces of evidence show that bacteria and host response interplay may form commensal microbiota composition though the precise mechanism of gastric inflammation leading to fecal microbiota variations is not indicated properly. Gastric microbiota and luminal pH changes may drive the community structure of gut microbiota [25].

Previous studies stated that colonization with non-*H. pylori* bacteria, gut commensals, changes the ‘resident’ gastric microbiota and the host equilibrium [11]. This article mainly reviewed the influence of intestinal microbiota on GC. Next, it discussed the potential mechanisms of intestinal microbiota in carcinogenesis. Moreover, the interactions between *H. pylori*, intestinal microbiota, and host in cancer induction were disserted. In the last part of this review, the effects of *H. pylori* and gut microbiota on metabolic pathways were discussed.

## Main text

### Gut microbiota in GC

*Lactobacillus*, *Lachnospiraceae*, *Escherichia-Shigella*, *Nitrospirae*, and *Burkholderia* are enhanced in GC patients compared with controls [8], confirming previous results with respect to the fact that *Lachnospiraceae* and *Lactobacillus* are abundantly found in GC [15, 26–28]. The findings pose a hypothesis; gastric colonization through non-*H*. *pylori* bacteria affects the GC risk, and many of them also colonize the intestine. The study by Ferreira et al. approved a notable decline in the abundance of *Helicobacter* and a significant increase in the genera *Achromobacter*, *Clostridium*, *Citrobacter*, *Rhodococcus*, and *Lactobacillus* in Portuguese patients with GC in comparison with chronic gastritis, the ORs were 20.5 (95 % CI 7.4–59), 5.7 (95 % CI 2.2–15), 9.9 (95 % CI 4.3–23), 4.2 (95 % CI 1.7–11), and 6.3 (95 % CI 2.9–14), respectively [11]. These members of microbiota are present as commensals in the intestinal mucosa but might be opportunistic pathogens [29, 30]. Evaluations on gastric microbiota show that *Lactobacillus* is highly abundant in progressive histological phases of gastric carcinogenesis and in GC patients [8, 10, 11, 26]. A study in Sweden found that *Lactobacillus* was one of the predominant genera in GC patients [15]. Increase of *Lactobacillus* sp from non-atrophic gastritis, to intestinal metaplasia and to GC was characterized in Mexican patients’ stomach microbiota using the microarray G3 PhyloChip [26]. In another study from Taiwan, *Lactobacillus* was a highly abundant species in GC patients [23]. Some commensal bacteria were overrepresented in GC. A large amount of *Klebsiella pneumoniae* and *Escherichia–Shigella* (belonging to *Enterobacteriaceae* taxa) was detected in GC patients’ gastric mucosa [7, 8]. A study in China showed that the dominant phyla in the feces of patients with gastric lesions were *Firmicutes*, *Bacteroidetes*, and *Proteobacteria* that accounted for the 99.05 % of all fecal bacteria, and *Bacteroides*, *Escherichia-Shigella*, *Prevotella_*9, and *Ruminococcus_*2 were the predominant genera [31]. Another study showed that there existed 12 bacterial genera enriched in GC, involving *Prevotella_9*, *Klebsiella*, *Lactobacillus*, *Escherichia*–*Shigella*, *Streptococcus*, *Veillonella*, *Alistipes*, *Bifidobacterium*, *Christensenellaceae_*R-7_group, *Ruminococcaceae_* UCG − 002, *Prevotella_*2, and *Parabacteroides*. *Enterobacteriaceae* and *Lachnospiraceae* had to be considered at the family level [32]. *Veillonella, Lactobacillus*, and *Streptococcus* of GC were increased, in terms of relative abundance, by 32.38-fold, 58.92-fold, and 15.93-fold, and *Tyzzerella_*3 and *Lachnospira* were declined by 8.85-fold and 3.37-fold, respectively. These reports show that the genera *Lactobacillus*, *Streptococcus*, *Veillonella*, *Tyzzerella_*3, and *Lachnospira* were employed to predict GC [32]. A study in China has shown that some *Actinobacteria* and *Firmicutes* species were considerably decreased in patients’ feces with esophageal cancer or GC compared to healthy individuals (P < 0.05) (Table 1) [33]. In comparison with normal and peritumoral tissues, *Prevotella copri* and *Bacteroides uniformis* showed a reduction while *Propionibacterium acnes*, *Streptococcus anginosus*, and *Prevotella melaninogenica* experienced an enhancement in tumor tissues [34]. Based on a recent study on various GC subtypes, *Patescibacteria*, *Bacteroidetes*, and *Fusobacteria* were enhanced in signet-ring cell carcinoma, while *Acidobacteria* and *Proteobacteria* showed an incremental trend in adenocarcinoma [35].

**Table 1:** The relationships of Gut microbiota with GC in the world

Gut microbiota (genera/species)	Related to increase ↑/decrease ↓ of GC	Country	ASR-Both sexes (GLOBOCAN 2018)	Study (Reference)
***Lactobacillus***, ***Lachnospiraceae***, ***Escherichia-Shigella***, ***Nitrospirae***, **and*Burkholderia***	↑	China	20.7	Wang et al., 2016 [8]
***Lactobacillus *and *Lachnospiraceae***	↑	South Korea	39.6	Eun et al., 2014 [28]
**genera *Achromobacter***, ***Clostridium***, ***Citrobacter***, ***Rhodococcus***, **and*Lactobacillus***	↑	Portugal	11. 0	Ferreira et al., 2018 [11]
***Lactobacillus***	↑	China	20.7	Coker et al., 2018 [10]
**genera *Streptococcus, Lactobacillus, Veillonella, and Prevotella***	↑	Sweden	3.3	Dicksved et al., 2009 [15]
***Lactobacillus *sp. and *Lachnospiraceae***	↑	Mexico City	5.6	Aviles-Jimenez et al., 2018 [26]
***Lactobacillus***	↑	Taiwan	-	Hsieh et al., 2018 [23]
***Escherichia–Shigella *and *Klebsiella pneumoniae *(belonging to Enterobacteriaceae taxa)**	↑	South Korea	39.6	Jo et al., 2016 [7]
**12 bacterial genera, including*Prevotella_9,Escherichia*–*Shigella***, ***Klebsiella,Lactobacillus,Streptococcus,Alistipes***, ***Veillonella,Bifidobacterium,Ruminococcaceae_UCG–002,Christensenellaceae_R-7_group,Parabacteroides,*and*Prevotella_2***	↑	China	20.7	Qi et al., 2019 [32]
**Species of*Firmicutes *and*Actinobacteria***	↓	China	20.7	Li et al., 2019 [33]

### Potential mechanisms of gut microbiota in carcinogenesis

Gut microbiota mechanisms contributing to carcinogenesis are not clear yet. Dysbiotic microbial community may increase the risk for gastric carcinoma by sustaining the gastric inflammatory process and triggering immune responses [11]. Also, microbial dysbiosis can increase inflammation and dysregulate the immune response, causing DNA mutations, hence, hastening the induction and/or progression of cancers. Such a mechanism may be due to interactions between fecal microbiota, *H. pylori* infection, and host responses [31]. It is well known that gastritis activity is correlated with *H. pylori* infection. Subsequent studies also confirmed such an association between gastritis activity and fecal microbiota. Intestinal flora could also increase the inflammatory responses in the mice stomach infected by *H. pylori*, promoting the development of neoplasia and gastric atrophy [36].

### Chronic inflammation

**Fig. 1:**
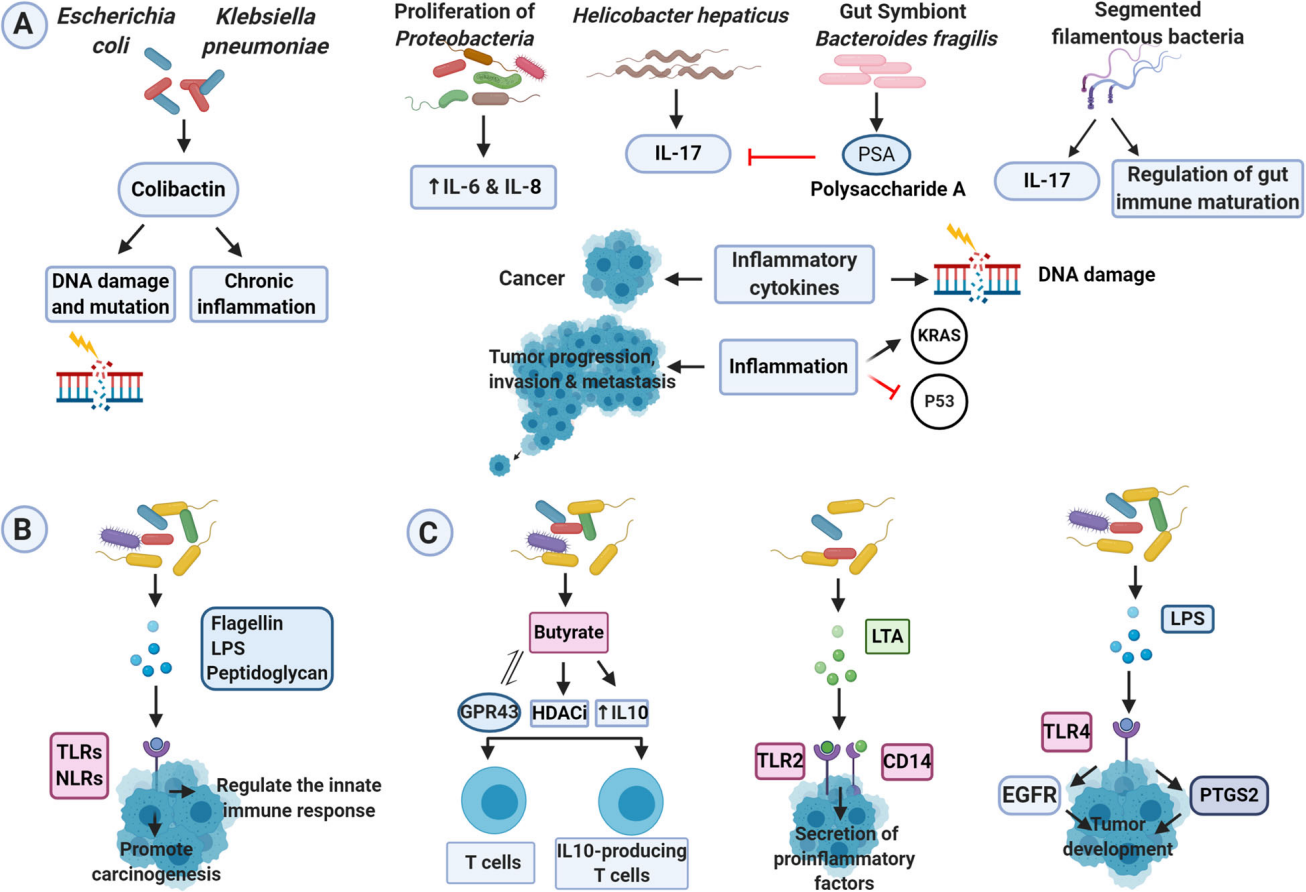
The mechanism of gut microbiota in carcinogenesis. **A**). Chronic inflammation: Inflammation accelerates the invasion, metastasis, and progression of the tumor. Inflammatory cytokines are directly involved in DNA damage and lead to inflammation-associated cancer. Inflammatory factors can inhibit tumor suppressor genes such as *p53*, and activate oncogenes such as *KRAS*. Colibactin produced by *Escherichia coli* and *Klebsiella pneumoniae* can cause DNA damage and mutations and trigger chronic inflammation. Proliferation of *Proteobacteria* is associated with an increase in IL-6 and IL-8. Polysaccharide A (PSA) expressed by symbiont *Bacteroides fragilis* can suppress IL-17 induced by *Helicobacter hepaticus*. Specific filamentous bacteria (SFB) are involved in the production of IL-17 and the regulation of gut immune maturation. **B**). Immune regulation: Dysbiosis of the gut microbiota is associated with cellular immunity and immune function, which affects GC development. The innate immune system can detect the structural components of gut microbiota such as flagellin, lipopolysaccharide (LPS), and peptidoglycans through Toll-like receptors (TLRs) and NOD-like receptors and regulate the innate immune response. **C**). Microbial metabolites: Butyrate through interaction with G protein-coupled receptor 43 (GPR43), up-regulating IL10 expression, and activating histone deacetylase inhibition (HDACi) can induce the differentiation of IL10-producing T cells and regulatory T cells. The Lipoteichoic acid (LTA) produced by the gut microbiota specifically binds to CD14 (cluster of differentiation 14) or TLR2 and causes the excessive secretion of proinflammatory agents, thus promotes malignant transformation. LPS produced by intestinal microbiota enhances tumor development by regulating the high expression of prostaglandin-endoperoxide synthase 2 (PTGS2) and activating the epidermal growth factor receptor (EGFR) signaling pathway

Inflammation aggravates the progression of tumor and hastens metastasis and invasion. Inflammatory cytokines damage DNA in the epithelium directly and induce inflammation-associated cancers [37]. The inflammation associated factors can stimulate oncogenes (e.g., *KRAS* mutation) and inactivate tumor-suppressor genes (e.g. *p53* mutation) [38, 39]. Investigations show that there exists an association between detrimental alterations in the composition of fecal microbiota and the increase in proinflammatory cytokines that induces the disease. *K. pneumoniae* and Colibactin-producing *Escherichia coli* can cause chronic inflammation, DNA damage, and mutation [40, 41]. Biagi et al. correlated the *Firmicutes* and *Bacteroidetes* reduction and *Proteobacteria* proliferation with IL-6 and IL-8 increases [42]. IL-11 and IL-6 can sensitize signal transducer and activator of transcription 3 (STAT3) activator, exerting a considerable effect on epithelial cells’ transformation [43]. The symbiont *Bacteroides fragilis*, which expresses polysaccharide A, is able to suppress proinflammatory IL-17 generation which is developed via *Helicobacter hepaticus* [44]. Intestinal commensals, segmented filamentous bacteria (SFB) in particular, were correlated with the gut immune maturation regulation and IL-17 generation (Fig. 1A). [45]. Lipoteichoic acid (LTA) also binds to CD14 or Toll-like receptor 2 (TLR2), inducing the excessive secretion of proinflammatory factors [46, 47].

### Microbial metabolites

Intestinal microbial dysbiosis has been associated with cellular immunity and immune function, which affects the GC development [48, 49]. NOD-like receptors (NLRs) and TLRs [50] can bridge this interplay, eventually promoting carcinogenesis in a chronic process. TLRs critically affect the innate immune system, assuming their capability in differentiating host molecules from microbial molecules. NLRs adjust the innate immune response, correspondingly activating inflammasome-mediated dysbiosis and modulating microbial composition (Fig. 1B). The gut microbiota can generate butyrate, which can differentiate regulatory T cells and IL10, generating T cells via the activation of histone deacetylase inhibition (HDACi), interactions with G protein-coupled receptor 43 (GPR43), and IL10 up-regulation [51]. LTA and short chain fatty acids (SCFAs) have opposite roles in carcinogenesis [52]. LTA accelerates malignant transformation. In contrast, SCFAs can mediate immunoregulation by Tregs, hence showing anti-carcinogenic and anti-inflammatory effects [51, 53, 54]. TLRs can develop gastrointestinal tract tumors by activating the STAT3 and NFKB signaling pathways [50]. The TLR4 activation, the receptor for LPS generated by the gut microbiome in the epithelial cells, can induce tumor development by up-regulating prostaglandin-endoperoxide synthase 2 (PTGS2) and activating the epidermal growth factor receptor (EGFR) signaling pathway in mice receiving AOM (Fig. 1C) [55]. In addition, the activation of the STAT3 signaling pathway up-regulates the expression of TLR2 in gastric epithelial cells, promoting tumor development in mice stomach [56].

### Bacterial genotoxins

It has been shown that intestinal bacteria are able to potentiate carcinogenesis by the specific toxins inducing DNA damage. As the disturbed microbes overgrow, they increase accumulating endotoxins and exotoxins, like cytolethal distending toxin from *Shigella dysenteriae*, cytolethal distending toxin and colibactin from *E. coli*, hydrogen peroxide, extracellular superoxide from *Enterococcus faecalis*, *Enterotoxigenic B. fragilis* toxin, a virulence factor activating the NFKB and WNT signaling pathways in epithelial cells [57–59], from *B. fragilis*, etc. These toxins directly or indirectly cause genomic instability, DNA damage, and the invasion of adenocarcinomas [40, 60–62]. Colibactin in *E. coli* can induce DNA damage, influence genomic instability [40, 63], and promote carcinogenesis. Colibactin-producing *K. pneumoniae* can induce chronic inflammation, DNA damage, and mutation [41]. Cytolethal distending toxin produced by the *Helicobacter* species and *E. coli* can induce DNA damage in mammalian cells [64–67]. The accumulation of base excision repair (BER) intermediates and unrepaired DNA cause genomic instability and carcinogenesis [68, 69].

### Mechanism of ***H. pylori*** in carcinogenesis

Genetic diversity can be defined as a leading characteristic of *H. pylori* strains due to intra-/intergenomic recombination and point mutations [70] which is correlated with the *H. pylori* pathogenicity, affecting the risk of malignancy [71]. *H. pylori* can regulate many signaling pathways, stimulate inflammation and immune responses, and trigger epithelial atrophy, achlorhydria, and dysplasia cancer [72]. *H. pylori* infection induces both innate and adaptive immune responses [73]. Upon recognition of *H. pylori* pathogen-associated molecular patterns (PAMPs) by the pattern recognition receptors (PRRs) of host cells, the initial stages of the innate immune responses are triggered [74]. As the main component of PRRs, TLRs have the ability to bind the LPS, CpG repeats, unmethylated nucleic acids, flagellin, double-stranded RNA, lipoteichoic acid, and lipoproteins of *H. pylori* [75]. Upon recognition of PAMPs, by activating activator protein (AP)-1, interferon regulatory factor (IRF), and NF-kB, TLRs manage to promote the expression of inflammatory mediators like TNF-α, IL-1, IL-2, IL-6, IL-8, IL-12, and IFN-γ [76, 77]. *H. pylori* is able to escape the recognition by the host PRRs of the innate immune response, which may lead to its long-term survival [78]. Concerning adaptive immunity, CD4^+^ T cells mediate the host immune response toward *H. pylori* infection [79]. CD4^+^ T cells have a higher abundance in GC samples than the peritumoral and normal tissues, while CD8^+^ T cells exhibited the opposite trend [80].

Inflammatory cytokines are highly accumulated in *H. pylori*-infected individuals’ stomach, including interferon-c, IL-1, TNF-α, IL1b, IL-7, IL-6, IL-8, IL-18, and IL-10. The oncogenic pathways’ activity containing ERK/MAPK, NF-kB, sonic hedgehog, PI3K/Akt, Ras, Wnt/beta-catenin, and STAT3 is up-regulated with *H. pylori* carrying cytotoxin-associated gene A (CagA). In contrast, with induced P53 mutations, tumor suppressor pathways become inactivate [81, 82]. *H. pylori* infection can induce methylations on E-cadherin CpG islands [83] and tumor-suppressor genes, consisting of those which encode a forkhead box transcriptional regulator (FOXD3) and the trefoil factor 2 (TFF2), which markedly increase the adenocarcinoma risk in the stomach [84]. The oncoprotein CagA and vacuolating cytotoxin A (VacA) are critical pathogenic factors of *H. pylori* infection [1, 85–87]. *H. pylori* expresses the CagA protein, which is a virulence factor that promotes cell proliferation by the activation of the signaling pathways of WNT, PI3K-AKT, and NF-kB [88–90], and reduces epithelial cell apoptosis by inhibiting TP53 [91]. Also, CagA has been approved to activate stemness features and stimulate the epithelial-mesenchymal transition (EMT) in gastric cells [92–96]. By acting on gastric epithelial cells, CagA promotes carcinogenesis through inflammation, proliferation induction, apoptosis inhibition, cell-cell bonding disruption, and loss of cell polarity [97]. The VacA toxin suppresses host immunity via inhibiting the activation of T-cells and inducing regulatory T cells [98–101]. The host immune response can be also modulated by VacA through inhibition of immune cell proliferation and stimulation of mast cells to produce proinflammatory cytokines; further promoting the development of gastritis associated with *H. pylori*, peptic ulceration, and GC [102]. It induces cell vacuolation [87, 103–105] and autophagy in human-derived gastric epithelial cells [106, 107] through directly affecting mitochondria [108–110], activating vascular endothelial growth factor [111, 112], up-regulating MAP kinase and ERK1/2 expression [113], up-regulating Wnt/beta-catenin pathway necessary for cell differentiation and growth [114], and suppressing GSK3 by the PI3K/Akt signaling pathway [115].

*H. pylori* virulence factors are involved in the host immune response [79]. The release of inflammatory mediators can activate Th1/Th17 cell responses and stimulate the production of TNF-α, IL-17, and IFN-γ [116]. Therefore, Th1/Th17 cells contribute to mediating the inflammatory response of patients suffering from *H. pylori* infection [116]. Inflammation may result in loss of acid-secreting parietal cells, hence, increasing the stomach pH, giving rise to declined *H. pylori* levels and incremental colonization of other bacteria [117]. *H. pylori* and chronic inflammation can promote the generation of both reactive nitrogen species (RNS) and reactive oxygen species (ROS), leading to DNA damages and induction of apoptosis or autophagy in the gastric epithelial cells [118]. Therefore, *H. pylori* can induce gastric carcinogenesis through genetic instability. Moreover, ROS induces DNA mutations in *H. pylori*, promoting its adaption to the host environments [119]. *H. pylori*-derived LPS can also cause specific impacts on GC cells through TLR4. *H. pylori* LPS stimulation activates the TLR4 signaling pathway in GC cells through affecting the expression of soluble factors or surface molecules which might help their evasion from CTLs or NK cells by IFN-γ–mediated cellular immune reaction [120]. Low induction of cellular immune response by *H. pylori* LPS can promote the host susceptibility toward GC development [120]. Based on Kidd et al., *H. pylori* LPS showed a specific mitogenic influence on gastric enterochromaffin-like cell neoplasia. LPS may exhibit poor virulence in evoking an inflammatory response while showing high potential in augmenting cell growth [121]. The enhanced LPS biosynthesis pathway of GC samples promoted microbiota-induced inflammations [122, 123].

### Interplays between ***H. pylori*** and gut microbiota

*H. pylori* infection can affect gut microbiota [122, 124]. It is associated with altered gastric microbiota and dysbiosis implicated in gastric disease pathogenesis [10, 11]. Wang et al.’s study showed that *H. pylori* infection was related with variations in human intestinal microbial composition and function in Chinese people [125]. Colonization of the stomach with IF (intestinal flora) promotes *H. pylori*-associated GC. IF effect in developing GC during *H. pylori* infection has been confirmed in previous studies [36]. Some bacteria, including *Bacteroides*, *Clostridium histolyticum*, *Prevotella* spp., and *Lactobacilli* have been associated with *H. pylori* infection in animal models and human trials [126–128]. *Prevotella copri* is known as a gut microbe that plays a role in the immune system. It was enriched significantly in *H. pylori*-positive patients. The continuous *H. pylori* colonization in the stomach brings about the host immune response [128, 129]. A study on a high-risk population showed that the genera *Gemella*, *Rhodococcus*, *Acidovorax*, and *Erysipelotrichaceae*_UCG-004 in fecal samples were associated with current *H. pylori* infection [31]. The relative abundances of dominant phyla in the gut of patients with positive *H. pylori* infection, involving *Firmicutes*, *Bacteroidetes*, and *Proteobacteria* are markedly different from those of individuals with negative *H. pylori* infection and may be associated with gastric lesions. The average relative abundances, for *Proteobacteria* and *Firmicutes*, showed high trends in the past *H. pylori* infection group (47.11, 20.53 %) in comparison with the negative group (23.44 and 9.05 %, respectively) although the *p*-values (0.068 and 0.246, respectively) revealed no meaningful variations [31]. A study on 1,123 Japanese adults approved more *Lactobacillus* in patients with *H. pylori*-infected patients suffering from severe atrophic gastritis [130]. According to Iino et al., infection with *H. pylori* initially affected the *Lactobacillus* species’ composition ratio in the gut microbiota prior to the progression of atrophic gastritis and proposed a greater *Lactobacillus* abundance in patients with *H. pylori* who suffered from severe atrophic gastritis [130]. Based on a German study, *H. pylori* increased the *lactobacilli* growth in fecal microbiome [126]. In the study by Yang et al., fecal microbiome was investigated in children with *H. pylori*-positive/-negative gastritis and healthy control groups. It was shown that at family and genus levels, the relative abundances of *Enterobacteriaceae* and *Bacteroidaceae* were common in gastritis with and without *H. pylori* infection, while the relative abundances of *Lactobacillaceae*, *Bifidobacteriaceae*, and *Lachnospiraceae* were high in healthy control group. To evaluate the *H. pylori* effect on gut microbiome among children, the fecal microbiome was analyzed in *H. pylori*-positive and -negative gastritis groups. The higher abundance of *Lactobacillales* and *Betaproteobacteria* and the lower abundance of *Alphaproteobacteria* were observed in *H. pylori*-positive group. Higher *Streptococcus* and *Collinsella* abundance was found at the genus and family levels in *H. pylori*-positive group relative to *H. pylori*-negative group [24]. The *H. pylori*-infected children also showed increased the number of gut microbiota including *Firmicutes*, *Proteobacteria*, *Prevotella*, and *Clostridium* compared with those without the infection [131]. In a study by Maldonado-Contreras, microbial community in *H. pylori*-positive subjects indicated an increase in the counts of *Proteobacteria*, *Acidobacteria*, and *Spirochaetes* [17]. In another study, the gut microbiota of individuals infected with *H*. *pylori* was reported to elevate in members of *Succinivibrio*, *Enterococcaceae*, *Coriobacteriaceae*, and *Rikenellaceae*. The greater abundance of these genera in individuals with *H. pylori* infection may be associated with the early stages of cancer development and *H. pylori* pathogenesis [132].

Various studies have shown that *H. pylori* infection affects the structure of the gut microbiota population. In contrast, some have reported that gut microbiota affects *H. pylori* colonization. As the diversity of intestinal flora microbiota increases, the level of *H. pylori* colonization decreases [36]. *H. pylori* eradication also incremented microbial diversity of the stomach [133]. Study of subjects at different gastric carcinogenesis histologic stages (gastritis, intestinal metaplasia, and GC) showed an inverse association between *H. pylori* load and microbial diversity of non-cancer gastric biopsies, whereas GC showed a lower diversity in comparison with other samples having the same *H. pylori* abundance; the difference could be assigned to antibiotic treatment [133]. *Lactobacillus casei* has been reported to inhibit the growth and colonization of *H. pylori* in the stomach [134]. Other studies have proved contradictory results about *Lactobacillus*. A study on *H. pylori* and *Lactobacillus* coisolates from humans did not prove a significant effect of *lactobacilli* on *H. pylori* strains [135]. A study on the gut microbiota of children with negative *H. pylori* showed the higher relative abundance of *bacteroidia, gammaproteobacteria*, *clostridia*, and *betaproteobacteria*, and a greater bacterial diversity and richness [136]. A study in China on children’s stool samples showed that at the genus and family levels, the lower abundances of *Erysipelotrichaceae*, *Pseudomonadaceae*, and *Megasphaera* were seen in *H. pylori*-positive group relative to *H. pylori*-negative group. It was also shown that the frequency of *Faecalibacterium* and *Roseburia* in the *H. pylori*-positive group was reduced compared to the healthy control group [24]. Many groups have employed sequencing-based and PCR-based methods to show that individuals with negative *H. pylori* have a very diverse gastric microbiota that is dominated by five predominant phyla: *Proteobacteria*, *Bacteroidetes*, *Firmicutes*, *Actinobacteria*, and *Fusobacteria* [16, 17, 137]. Conversely, *H. pylori* is the utmost abundant bacterium in the stomach and involves the 97 and 72 % of all sequence reads among the subjects with positive *H. pylori* [16, 137]. In a study by Maldonado-Contreras, the microbial community in individuals with positive *H. pylori* was known by a decline in *Bacteroidetes*, *Actinobacteria*, and *Firmicutes* counts [17]. Bik et al., reported that the individuals with negative *H. pylori* carry the higher abundant phyla of *Bacteroidetes*, *Firmicutes*, and *Actinobacteria* [16]. Conversely, a study from China showed that the relative abundance of *Bacteroidetes* was greatly reduced from *H. pylori* negative to past infection community (66.16 %, 33.01 %, respectively; *p* = 0.007). *Rhodococcus* and *Acidovorax* had slightly lower average relative abundance at the genus level in patients that are currently infected with *H. pylori* compared with others that are not currently infected (*p* = 0.017 and 0.016, respectively). It was also shown that the average relative abundance of the two genera (the phylum of *Bacteroidetes*; *Barnesiella* and *Parabacteroides*) was decreased among the groups having the different status of *H. pylori* infection (negative: 1.15 and 2.44 %, past infection: 0.58 and 1.27 %, respectively) [31].

### ***H. pylori*** and gut microbiota interaction in cancer

The interactive associations between *H. pylori* and other gastric bacteria have not been completely understood [102]. *H. pylori* infection has been linked to altered gastrointestinal microbiota and dysbiosis, all of which have been linked to the pathogenesis of gastric diseases [10, 11]. It is not, however, clear whether infection with *H. pylori* itself approves the growth of unwanted microorganisms or an altered microbiota brings about beneficial situations for the colonization of *H. pylori* [123]. Probably, there is a multifaceted interaction, where the *H. pylori* colonization contributes to the growth of some microbes and vice versa. It is likely that dysbiosis alters gastric mucosa which is highly desired for the colonization of *H. pylori* [124].

Some researchers believe that *H. pylori* is more of a latent or opportunistic pathogen than a pathogenic bacterium and can be considered a commensal organism. This is important because we know that the majority of the world’s population is infected with *H. pylori* and colonization occurs with bacteria that carry or do not carry critical virulence factors at an early age. However, it should be noted that severe gastrointestinal diseases or complications occur mainly in adults with age > 40 years and only in < 10 % of infected individuals. This low incidence clearly showed that *H. pylori* is more of a latent or opportunistic pathogen than a pathogenic bacterium, and that virulence factors play little role in the outcome of the disease [138]. Long-term colonization of *H. pylori* and its interaction with other gastric microbiota appear to alter gastric mucosal dysbiosis and lead to the development of severe gastrointestinal disease, including GC, by inducing persistent and long-term inflammatory responses [31, 124].

It can be speculated that the alterations of gut microbiota induced by *H. pylori* may affect the development of GC since the composition of microbiota stimulates immune responses at a systemic and local level; moreover, the development of GC is affected by inflammatory signaling [31]. The interaction between gut microbiota and *H. pylori* is not known yet and literature reveals inconsistent results [132]. The gastritis activity is perceived for its tight correlation with *H. pylori* infection, which is further approved by similar changes observed in the fecal microbiota from the subjects with non-active gastritis and past infection. Additionally, the same alteration tendencies were found for major genera or phyla, including reduced *Bacteroidetes* abundance and increased *Proteobacteria* or *Firmicutes* abundances, with gastric lesion severity and *H. pylori* infection status (particularly the status of past infection). Furthermore, it states that changes in intestinal microbiota may develop precancerous gastric lesions related to *H. pylori* and carcinogenesis [31]. It has been suggested that lactic acid-producing bacteria may promote gastric inflammatory reactions induced by *H. pylori* [24]. Lactic acid bacteria promote immune tolerance, providing the platform for colonization of other carcinogenic bacteria [139].

By modulating the acidity of the stomach, *H. pylori* could change the gastric microbiome profiles, promoting *H. pylori*-associated disorders. Alterations in the gastric environment that decline acid secretion can encourage the growth of NOC-producing bacteria, thus elevating the chance of gastric carcinogenesis [102]. Th1/Th17 cells contribute to the inflammatory response of *H. pylori*-infected patients [116, 140] Inflammation increases gastric pH, which decreases *H. pylori* levels and increases non-*H. pylori* bacteria in the stomach [117]. There is a significant difference in the microbial profiles and composition of early and advanced GC, reflecting the changes related to GC progression. The gastric microbiome alterations in early GC stages could be assigned to host genetic changes, *H. pylori* infection, bacterial virulence, and adaptation to the environment. Constrained principal coordinate analyses indicated the influence of *H. pylori* and *cagA* and *vacA* genotypes on the gastric microbiome structure. The detected microbial fingerprint can be regarded as a biomarker for clinical evaluation of GC risk among high-risk cases [141].

### Effect of ***H. pylori*** and gut microbiota on metabolic pathways and carcinogenesis

Gut microbiota changes correlate with different inflammatory and metabolic illnesses. Little is known about the effect of *H*. *pylori* on downstream gut microbiota though many studies have examined the correlation between gastric microbiota and *H*. *pylori* [132]. Microbiome alterations are often followed by variations in microbial functions. The relative abundance of 19 gut microbial pathways differs significantly between *H. pylori*-negative and *H. pylori*-positive subjects [142]. Persistent *H. pylori* infection can induce detrimental inflammatory processes besides the impact on host microbes [143]. Epidemiological studies show that *H. pylori* infection is related with the lower levels of vitamin B12 (VB12) in the blood [144, 145]. The *H. pylori* infection-related intestinal microbiota dysbiosis can influence the VB12 production. VB12 is a cobalt corrinoid. As humans cannot produce VB12, it is generated exclusively by the microorganisms, especially anaerobes [146]. In the study of Wang et al., it was observed that the levels of plasma VB12 and gut microbial VB12 biosynthesis were meaningfully lower in the subjects with positive *H. pylori* in comparison to the subjects with negative *H. pylori* (*p* < 0.05, Wilcoxon test). Lower VB12 biosynthesis module was linked to the lower levels of VB12 concentrations in subjects with *H. pylori* infection, manifesting that *H. pylori* infection-related gut microbiota dysbiosis enhances the VB12 deficiency risk. This shows that some changes in gut microbial species and functions correlate with *H. pylori* infection, suggesting that the gut microbial shift in the patients with *H. pylori* infection may raise VB12 deficiency indirectly [125]. In addition, previous studies have inferred that gastric sinusitis, induced by *H. pylori* infection, may develop type B chronic gastritis, followed by reduced the secretion of gastric acid, leading to VB12 malabsorption [145, 147]. Thus, both the absorption capacity and production of VB12 can be attenuated by *H. pylori* infection, augmenting the VB12 deficiency risk. Low serum vitamin B12 levels are significantly correlated to the elevated risk of non-cardia gastric adenocarcinoma (NCGA) [148]. *H. pylori* infection has been also related to food-bound vitamin B12 malabsorption [149, 150] possibly because of the atrophic gastritis induction which is accompanied by achlorhydria (increased gastric pH). Furthermore, vitamin B12 absorption needs acid-producing gastric mucosa, allowing for vitamin B12 cleavage from its binding proteins [151]. As a result, any stimulus inducing chronic atrophic gastritis can enhance the risk of NCGA, disturb vitamin B12 absorption, and thus, declines its serum concentrations [148]. Since vitamin B12 uptake necessitates intact gastric mucosa for acid production, the findings proposed vitamin B12 as a potential serologic marker of NCGA-preceding atrophic gastritis [148].

**Fig. 2:**
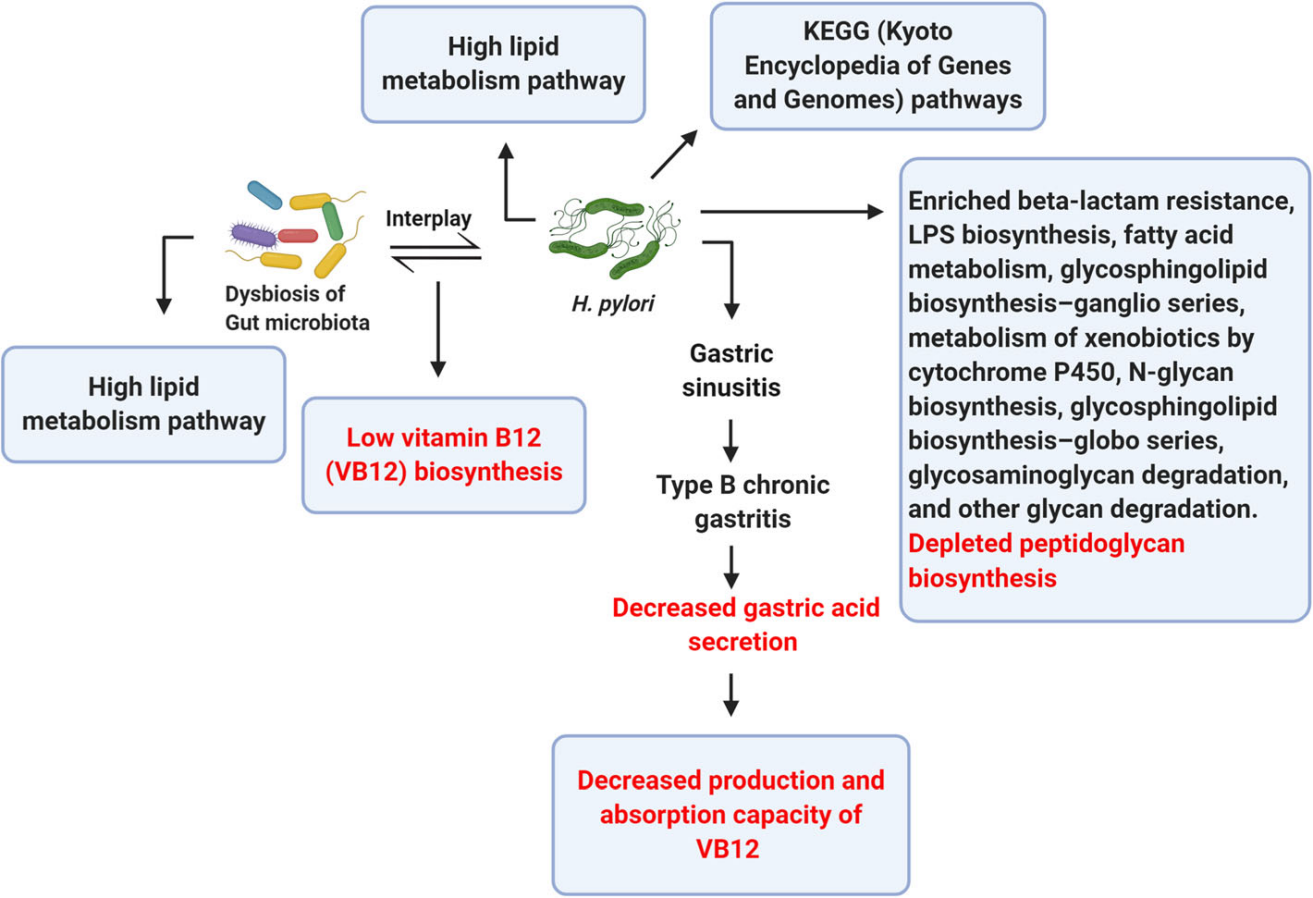
The *H. pylori* infection-related dysbiosis of gut microbiota correlates with the low levels of vitamin B12 (VB12) production. Gastric sinusitis caused by *H. pylori* infection is also associated with the decreased production and absorption of vitamin VB12. KEGG (Kyoto Encyclopedia of Genes and Genomes) pathways and pathways related to metabolisms (lipopolysaccharide (LPS) biosynthesis, beta-lactam resistance, glycosphingolipid biosynthesis–globo series, glycosphingolipid biosynthesis–ganglio series, fatty acid metabolism, xenobiotic metabolism by cytochrome P450, N-glycan biosynthesis, glycosaminoglycan degradation, and other glycan degradation) are increased in *H. pylori* infection, and peptidoglycan biosynthesis pathways are decreased in infection with this bacterium. Both *H. pylori* infection and gut microbiota dysbiosis are associated with the high metabolism of lipid

A recent study on children has shown that altered intestinal microbiota, gastritis, and *H. pylori* interact with each other. Possibly, *H. pylori* changes the gut micro-environmental cues, like pH alterations that cause this compositional shift between native communities to compensate. This compensation will be translated into distinctive functional genes contributing to crucial metabolic pathways [132]. It has also been shown that gut microbiome influenced by gastritis and *H. pylori* infection changes the body’s basal metabolic function [24]. Seventeen KEGG pathways revealed notable variations in *H. pylori*-infected group and healthy control group. The results of this study manifested the meaningful increase of activity in children’s metabolic pathways, who are *H. pylori*-positive [24]. However, peptidoglycan biosynthesis was depleted in the *H. pylori*-positive group as metabolism-related pathways (fatty acid metabolism, LPS biosynthesis, beta-lactam resistance, xenobiotics metabolism by cytochrome P450, glycosphingolipid biosynthesis–ganglio series, glycosphingolipid biosynthesis–globo series, N-glycan biosynthesis, and glycosaminoglycan degradation) were enriched in the *H. pylori*-positive group [24]. *H. pylori* is dependent on unsaturated fatty acid (UFA) biosynthesis to maintain its membrane function and structure [152]. The microbial UFA level is meaningfully increased in the blood of the patients developing *H. pylori*-induced peptic ulceration [153]. Based on these results, it can be said that *H. pylori* is associated with the high metabolism of lipid. According to the microbiome’s functional analysis, lipid metabolism pathway was increased in the gastritis group, showing that gut microbiome similarly affects *H. pylori*-induced gastritis (Fig. 2) [24]. UFA biosynthesis plays a decisive role in the integrity of membrane structure and function. *H. pylori* can grow at anaerobic conditions [152], allowing for *H. pylori* persistence and induction of carcinogenic consequences within the gastric niche.

### Effect of gut microbiota on cell metabolites and carcinogenesis

**Fig. 3:**
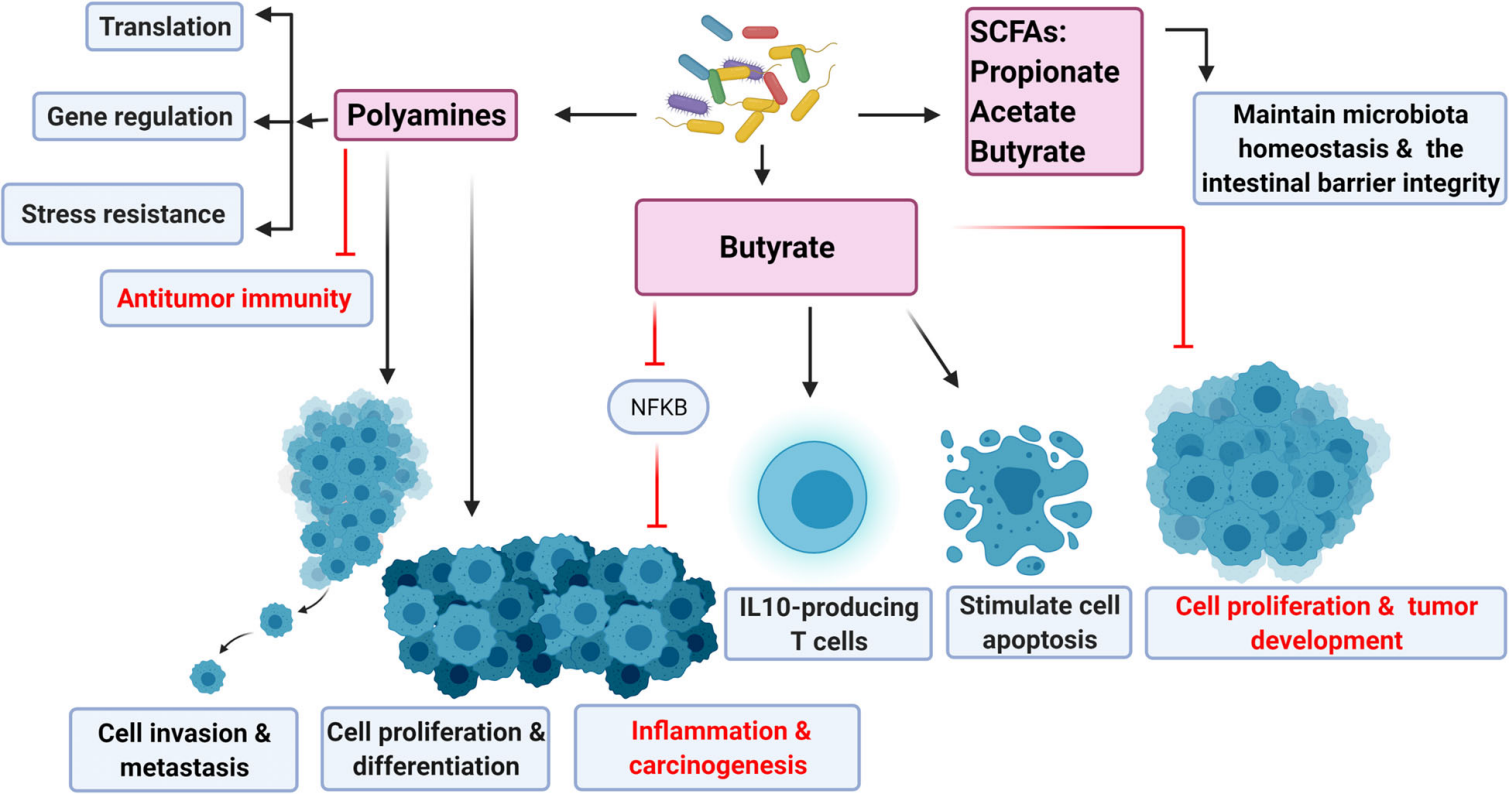
Gut microbiota produce various metabolites that are involved in the development and progression of cancer. Polyamines play important roles in translation, gene regulation, stress resistance, antitumor immunity suppression, cell proliferation, invasion, and metastasis. Short chain fatty acids (SCFAs) generated by intestinal microbiota, such as butyrate, acetate, and propionate can maintain microbiota homeostasis and the intestinal barrier integrity. Butyrate produced by gut microbiota can suppress inflammation and carcinogenesis by blocking the signaling pathway of NFKB activation. Butyrate can induce the differentiation of regulatory T cells and IL10-producing T cells. Butyrate also acts as a histone deacetylase and leads to the inhibition of cell proliferation, stimulation of apoptosis, and suppression of tumor development

Intestinal bacteria generate different metabolites affecting the progression and development of gastrointestinal tract tumors [154]. Polyamines, generated by gut bacteria and host cells, largely influence different pathologic and biologic processes, such as translation, stress resistance, gene regulation, and cell differentiation and proliferation [155]. Polyamines suppress antitumor immunity and promote cancer cells’ proliferation, invasion, and metastasis [156]. SCFAs are dietary fiber fermentation products generated by intestinal microbiota, such as propionate, acetate, and butyrate. They can maintain microbiota homeostasis and the intestinal barrier integrity and suppress inflammation and cancer [157]. SCFAs such as butyrate, generated by the gut microbiota, may inhibit carcinogenesis and inflammation through blocking the activation of the NFKB signaling pathway, and differentiating IL10-producing T cells and regulatory T cells [51, 158, 159]. Moreover, butyrate can act as a histone deacetylase inhibitor to suppress the proliferation of the cells, induce apoptosis, and suppress the development of the tumor [160–162]. In contradiction, low butyrate concentrations may potentiate the tumor growth that, in a mouse model, suppresses DNA mismatch repair deficiencies (Fig. 3) [163].

Functional analysis of the gastric microbiome indicated a significant reduction in the production of urease and bacterial flagella synthesis at early GC stages, whereas fructose glycolysis and glycosides hydrolysis showed an enhancement. The frequency of glucose-6-phosphate dehydrogenase exhibited a decrease, reflecting a decrement in carbohydrate degradation. The relative frequency of 6-phosphofructokinase (COG205) showed a drastic reduction in advanced GC cases [141]. Numerous bacteria (e.g. *Nitrospirae, Lactobacillus*, *Neisseria*, *Staphylococcus*, *Haemophilus*, *Clostridium*, and *Veillonella)* promote gastric carcinogenesis through stimulation of the N-nitroso compounds (NOCs) production [8, 164]. Higher levels of lactic acid bacteria were found in GC patients [165]. These bacteria may enhance the GC risk by several mechanisms such as elevated generation of ROS, NOCs, and lactate in addition to inducing EMT and immune tolerance [102]. *In vitro* and *in vivo* investigations suggested the stimulating role of lactic acid bacteria in ROS generation which may lead to DNA damage. Enhanced the formation of NOCs can promote mutagenesis, angiogenesis, and the expression of protooncogenes, resulting in apoptosis inhibition [139, 166]. Lactic acid bacteria-produced lactate is a robust energy source for cancer cells [167] with a regulatory role in various carcinogenesis issues such as tumor angiogenesis and metastasis [168]. These bacteria are capable of promoting EMT with contributive roles in tumor invasion and metastasis [169] through induction of multipotency state [139].

### Microbiome-based GC therapy

Conventional GC therapies such as surgery, chemotherapy, and radiotherapy have not shown high efficacy [102]. *H. pylori* eradication could be an effective approach to reduce the GC risk. Antibiotic treatment of *H. pylori* has been shown to alter the gastric microbiome composition [133, 164]. Regarding the increasing rate of antibiotic resistance of *H. pylori*, novel *H. pylori* eradication strategies are urgently required. Some probiotics have shown promises in the prevention of antibiotic-induced adverse impacts, an increase in *H. pylori* eradication rate, and the reduction of fluctuations in the gut microbiome profiles [170]. *Lactobacillus* supplementation can effectively eradicate *H. pylori* [171, 172] and reduce the chance of GC development [102]. Some *Lactobacillus* strains mitigated *H. pylori* by inhibiting its adhesion to epithelial cells, production of organic acids or bacteriocins, and suppression of mucosal inflammation [173, 174]. *Lactobacillus acidophilus* and *Lactobacillus bulgaricus* can decrement the *H. pylori* adhesion to gastric mucosal cells [175], *L. bulgaricus* showed inhibitory impacts on IL-8 production of mucosal cells by modulation of the TLR4/IkBa/NF-kB pathways [175]. A probiotic mixture containing *Lactobacillus* and *Bifidobacterium* showed helpful influences against *H. pylori*, at low side effects [176]. A combination of *Bacillus cereus*, *Enterococcus faecalis, L. acidophilus*, and *Bifidobacterium infantis* increased the host immunity and declined inflammation among GC cases undergoing gastrectomy [177].

Despite the traditional carcinogenic role of bacteria, new studies have revealed their anticancer features. The anticancer properties of bacteria can be assigned to various mechanisms such as colonization in tumors, releasing active agents, a carrier for anticancer drugs delivery, suppression of vital nutrients for tumor metabolism and proliferation, reinforcement of host immunity, and biofilm formation [102, 178, 179]. The KLA peptide is a pro-apoptosis peptide KLAKLAKKLAKLAK with anticancer activities through apoptosis induction by disrupting mitochondrial membrane; it however showed poor membrane permeability [180]. HPRP-A1 (*H. pylori* ribosomal protein) and its enantiomer HPRP-A2 (15-mer cationic peptides) can be derived from the N-terminus of *H. pylori* ribosomal protein L1 [181]. HPRP-A1 and HPRP-A2 have exhibited powerful antimicrobial and anticancer features. HPRP-A1—a membrane-active peptide—is capable of disrupting the tumor cell membrane. It is largely employed in drug delivery to cancer cells [182]. HPRP-A1 can facilitate the entry of KLA peptides to cancer cells, hence, promoting tumor cell death [183]. Apoptosis induction of HPRP-A2 in the GC cells is achieved via elevation of ROS production; activation of caspases (3, 8, and 9); reduction of mitochondrial membrane potential, and cell cycle arrest within the G1 phase [184].

## Conclusions

Mechanistic studies evaluating how gut microbes regulate health and promote gastrointestinal cancers are still at the early stage. Nevertheless, researchers have determined that gut microbiota are in close relation with humans and markedly influence GC and human health. Researchers have taken some steps to regulate gut microbes. The objectives are multifaceted, including the regulation of human metabolism, immune, and inflammatory reaction, as well as inhibiting carcinogenesis and cancer progression. Significant advances have been made in understanding the interaction between *H. pylori* and intestinal microbiota in the development of gastritis and cancer. However, there have been controversies in the findings of different studies which seem to be due to environmental differences (e.g., diet, etc.) or genetic differences of the host. Detailed studies in well-defined human populations are still required to compare the composition differences of the gut microbiome in different anatomical regions of the stomach of individuals developing *H. pylori* infection with and without neoplastic lesions. Future investigations are recommended to assess the effect of the gut microbiome composition in various anatomical stomach regions on the risk of cancer. These could be carried out by the site-specific topographical mapping of the microbiota in the absence or presence of *H. pylori* and by assessing variations with respect to the states of the disease along the gastric carcinogenesis cascade [185]. Deeper and better understanding of the relationship between *H. pylori-*related precancerous gastric lesions and gut microbiota, and the complicated interaction between them can have a significant impact on the design of new strategies for the prevention, diagnosis and treatment of GC.

## Data Availability

Data sharing is not applicable to this article as no datasets were generated or analysed during the current study.

## References

[1] Bakhti SZ, Latifi-Navid S, Safaralizadeh R (2020). *Helicobacter pylori***-related risk predictors of gastric cancer: The latest models, challenges, and future prospects**. Cancer medicine.

[2] Abdi E, Latifi-Navid S, Zahri S, Yazdanbod A, Pourfarzi F (2019). Risk factors predisposing to cardia gastric adenocarcinoma: Insights and new perspectives. Cancer medicine.

[3] Bakhti SZ, Latifi-Navid S, Zahri S (2020). **Unique constellations of five polymorphic sites of***Helicobacter pylori vacA***and***cagA***status associated with risk of gastric cancer**. Infection genetics evolution: journal of molecular epidemiology evolutionary genetics in infectious diseases.

[4] Abdi E, Latifi-Navid S, Abedi Sarvestani F, Esmailnejad MH: **Emerging Therapeutic Targets for Gastric Cancer from a Host**-Helicobacter pylori **Interaction Perspective**. *Expert opinion on therapeutic targets* 2021. 10.1080/14728222.2021.1971195 34410200

[5] Amieva M, Peek RM. Jr.: **Pathobiology of** *Helicobacter pylori***-Induced Gastric Cancer**. Gastroenterology. 2016;150(1):64–78. 10.1053/j.gastro.2015.09.004 PMC4691563 26385073

[6] Hu Y-L, Pang W, Huang Y, Zhang Y, Zhang C-J (2018). The Gastric Microbiome Is Perturbed in Advanced Gastric Adenocarcinoma Identified Through Shotgun Metagenomics. Front Cell Infect Microbiol.

[7] Jo HJ, Kim J, Kim N, Park JH, Nam RH, Seok YJ, Kim YR, Kim JS, Kim JM, Kim JM (2016). **Analysis of gastric microbiota by pyrosequencing: minor role of bacteria other than***Helicobacter pylori***in the gastric carcinogenesis**. Helicobacter.

[8] Wang L, Zhou J, Xin Y, Geng C, Tian Z, Yu X, Dong Q (2016). Bacterial overgrowth and diversification of microbiota in gastric cancer. Eur J Gastroenterol Hepatol.

[9] Flemer B, Lynch DB, Brown JM, Jeffery IB, Ryan FJ, Claesson MJ, O’Riordain M, Shanahan F, O’Toole PW (2017). Tumour-associated and non-tumour-associated microbiota in colorectal cancer. Gut.

[10] Coker OO, Dai Z, Nie Y, Zhao G, Cao L, Nakatsu G, Wu WK, Wong SH, Chen Z, Sung JJ (2018). Mucosal microbiome dysbiosis in gastric carcinogenesis. Gut.

[11] Ferreira RM, Pereira-Marques J, Pinto-Ribeiro I, Costa JL, Carneiro F, Machado JC, Figueiredo C (2018). Gastric microbial community profiling reveals a dysbiotic cancer-associated microbiota. Gut.

[12] Gopalakrishnan V, Spencer CN, Nezi L, Reuben A, Andrews M, Karpinets T, Prieto P, Vicente D, Hoffman K, Wei S (2018). Gut microbiome modulates response to anti–PD-1 immunotherapy in melanoma patients. Science.

[13] Lofgren JL, Whary MT, Ge Z, Muthupalani S, Taylor NS, Mobley M, Potter A, Varro A, Eibach D, Suerbaum S (2011). **Lack of commensal flora in***Helicobacter pylori***-infected INS-GAS mice reduces gastritis and delays intraepithelial neoplasia**. Gastroenterology.

[14] Sanduleanu S, Jonkers D, De Bruine A, Hameeteman W, Stockbrügger R (2001). **Non-***Helicobacter pylori***bacterial flora during acid‐suppressive therapy: differential findings in gastric juice and gastric mucosa**. Aliment Pharmacol Ther.

[15] Dicksved J, Lindberg M, Rosenquist M, Enroth H, Jansson JK, Engstrand L (2009). Molecular characterization of the stomach microbiota in patients with gastric cancer and in controls. Journal of medical microbiology.

[16] Bik EM, Eckburg PB, Gill SR, Nelson KE, Purdom EA, Francois F, Perez-Perez G, Blaser MJ, Relman DA: **Molecular analysis of the bacterial microbiota in the human stomach**. *Proceedings of the National Academy of Sciences* 2006, **103**(3):732–737. 10.1073/pnas.0506655103 PMC1334644 16407106

[17] Maldonado-Contreras A, Goldfarb KC, Godoy-Vitorino F, Karaoz U, Contreras M, Blaser MJ, Brodie EL, Dominguez-Bello MG (2011). **Structure of the human gastric bacterial community in relation to***Helicobacter pylori***status**. ISME J.

[18] Correa P (1992). Human gastric carcinogenesis: a multistep and multifactorial process—first American Cancer Society award lecture on cancer epidemiology and prevention. Cancer research.

[19] Ge Z, Feng Y, Taylor NS, Ohtani M, Polz MF, Schauer DB, Fox JG (2006). **Colonization dynamics of altered Schaedler flora is influenced by gender, aging, and***Helicobacter hepaticus***infection in the intestines of Swiss Webster mice**. Appl Environ Microbiol.

[20] Stearns JC, Lynch MD, Senadheera DB, Tenenbaum HC, Goldberg MB, Cvitkovitch DG, Croitoru K, Moreno-Hagelsieb G, Neufeld JD (2011). Bacterial biogeography of the human digestive tract. Scientific reports.

[21] Roos S, Engstrand L, Jonsson H (2005). *Lactobacillus gastricus***sp. nov.**, *Lactobacillus antri***sp. nov.**, *Lactobacillus kalixensis***sp. nov. and***Lactobacillus ultunensis***sp. nov., isolated from human stomach mucosa**. Int J Syst Evol MicroBiol.

[22] Ryan KA, Jayaraman T, Daly P, Canchaya C, Curran S, Fang F, Quigley EM, O’Toole PW: **Isolation of lactobacilli with probiotic properties from the human stomach**. *Letters in applied microbiology* 2008, **47**(4):269–274. 10.1111/j.1472-765x.2008.02416.x 19241519

[23] Hsieh Y-Y, Tung S-Y, Pan H-Y, Yen C-W, Xu H-W, Lin Y-J, Deng Y-F, Hsu W-T, Wu C-S, Li C (2018). Increased abundance of Clostridium and Fusobacterium in gastric microbiota of patients with gastric cancer in Taiwan. Scientific reports.

[24] Yang L, Zhang J, Xu J, Wei X, Yang J, Liu Y, Li H, Zhao C, Wang Y, Zhang L (2019). *Helicobacter pylori***infection aggravates dysbiosis of gut microbiome in children with gastritis**. Frontiers in Cellular Infection Microbiology.

[25] Cremer J, Arnoldini M, Hwa T: **Effect of water flow and chemical environment on microbiota growth and composition in the human colon**. *Proceedings of the National Academy of Sciences* 2017, **114**(25):6438–6443. 10.1073/pnas.1619598114 PMC5488924 28588144

[26] Aviles-Jimenez F, Vazquez-Jimenez F, Medrano-Guzman R, Mantilla A, Torres J (2014). Stomach microbiota composition varies between patients with non-atrophic gastritis and patients with intestinal type of gastric cancer. Scientific reports.

[27] Riley DR, Sieber KB, Robinson KM, White JR, Ganesan A, Nourbakhsh S, Hotopp JCD (2013). Bacteria-human somatic cell lateral gene transfer is enriched in cancer samples. PLoS Comput Biol.

[28] Eun CS, Kim BK, Han DS, Kim SY, Kim KM, Choi BY, Song KS, Kim YS, Kim JF (2014). Differences in gastric mucosal microbiota profiling in patients with chronic gastritis, intestinal metaplasia, and gastric cancer using pyrosequencing methods. Helicobacter.

[29] Kelly CP, LaMont JT (2008). *Clostridium difficile***—more difficult than ever**. N Engl J Med.

[30] Rajilić-Stojanović M, de Vos WM (2014). The first 1000 cultured species of the human gastrointestinal microbiota. FEMS MicroBiol Rev.

[31] Gao J-J, Zhang Y, Gerhard M, Mejias-Luque R, Zhang L, Vieth M, Ma J-L, Bajbouj M, Suchanek S, Liu W-D (2018). **Association between gut microbiota and***Helicobacter pylori***-related gastric lesions in a high-risk population of gastric cancer**. Front Cell Infect Microbiol.

[32] Qi Y-f, Sun J-n, Ren L-f, Cao X-l, Dong J-h, Tao K (2019). Guan X-m, Cui Y-n, Su W: **Intestinal microbiota is altered in patients with gastric cancer from Shanxi province, China**. Digestive diseases sciences.

[33] Li NN, Bai CM, Zhao L, Ge YP (2019). Gut Microbiome Differences between Gastrointestinal Cancer Patients and Healthy People. Zhongguo yi xue ke xue yuan xue bao Acta Academiae Medicinae Sinicae.

[34] Liu X, Shao L, Liu X, Ji F, Mei Y, Cheng Y, Liu F, Yan C, Li L, Ling Z (2019). Alterations of gastric mucosal microbiota across different stomach microhabitats in a cohort of 276 patients with gastric cancer. EBioMedicine.

[35] Ravegnini G, Fosso B, Saverio VD, Sammarini G, Zanotti F, Rossi G, Ricci M, D’Amico F, Valori G, Ioli A (2020). Gastric Adenocarcinomas and Signet-Ring Cell Carcinoma: Unraveling Gastric Cancer Complexity through Microbiome Analysis—Deepening Heterogeneity for a Personalized Therapy. Int J Mol Sci.

[36] Lertpiriyapong K, Whary MT, Muthupalani S, Lofgren JL, Gamazon ER, Feng Y, Ge Z, Wang TC, Fox JG (2014). **Gastric colonisation with a restricted commensal microbiota replicates the promotion of neoplastic lesions by diverse intestinal microbiota in the***Helicobacter pylori***INS-GAS mouse model of gastric carcinogenesis**. Gut.

[37] Hattori N, Ushijima T (2016). Epigenetic impact of infection on carcinogenesis: mechanisms and applications. Genome medicine.

[38] Hussain SP, Amstad P, Raja K, Ambs S, Nagashima M, Bennett WP, Shields PG, Ham A-J, Swenberg JA, Marrogi AJ (2000). Increased p53 mutation load in noncancerous colon tissue from ulcerative colitis: a cancer-prone chronic inflammatory disease. Cancer research.

[39] Raponi M, Winkler H, Dracopoli NC (2008). KRAS mutations predict response to EGFR inhibitors. Curr Opin Pharmacol.

[40] Cuevas-Ramos G, Petit CR, Marcq I, Boury M, Oswald E, Nougayrède J-P: Escherichia coli **induces DNA damage in vivo and triggers genomic instability in mammalian cells**. *Proceedings of the National Academy of Sciences* 2010, **107**(25):11537–11542. 10.1073/pnas.1001261107 PMC2895108 20534522

[41] Kaur CP, Vadivelu J, Chandramathi S (2018). Impact of Klebsiella pneumoniae in lower gastrointestinal tract diseases. Journal of digestive diseases.

[42] Biagi E, Nylund L, Candela M, Ostan R, Bucci L, Pini E, Nikkïla J, Monti D, Satokari R, Franceschi C (2010). Through ageing, and beyond: gut microbiota and inflammatory status in seniors and centenarians. PloS one.

[43] Putoczki TL, Thiem S, Loving A, Busuttil RA, Wilson NJ, Ziegler PK, Nguyen PM, Preaudet A, Farid R, Edwards KM (2013). Interleukin-11 is the dominant IL-6 family cytokine during gastrointestinal tumorigenesis and can be targeted therapeutically. Cancer cell.

[44] Mazmanian SK, Round JL, Kasper DL (2008). A microbial symbiosis factor prevents intestinal inflammatory disease. Nature.

[45] Chung H, Pamp SJ, Hill JA, Surana NK, Edelman SM, Troy EB, Reading NC, Villablanca EJ, Wang S, Mora JR (2012). Gut immune maturation depends on colonization with a host-specific microbiota. Cell.

[46] Ginsburg I (2002). Role of lipoteichoic acid in infection and inflammation. The Lancet infectious diseases.

[47] Hermann C, Spreitzer I, Schröder NW, Morath S, Lehner MD, Fischer W, Schütt C, Schumann RR, Hartung T (2002). Cytokine induction by purified lipoteichoic acids from various bacterial species–Role of LBP, sCD14, CD14 and failure to induce IL-12 and subsequent IFN‐γ release. Eur J Immunol.

[48] Vijay-Kumar M, Gewirtz A (2009). Flagellin: key target of mucosal innate immunity. Mucosal immunology.

[49] Hayashi F, Smith KD, Ozinsky A, Hawn TR, Eugene CY, Goodlett DR, Eng JK, Akira S, Underhill DM, Aderem A (2001). The innate immune response to bacterial flagellin is mediated by Toll-like receptor 5. Nature.

[50] Rakoff-Nahoum S, Medzhitov R (2009). Toll-like receptors and cancer. Nat Rev Cancer.

[51] Smith PM, Howitt MR, Panikov N, Michaud M, Gallini CA, Bohlooly-Y M, Glickman JN, Garrett WS (2013). The microbial metabolites, short-chain fatty acids, regulate colonic Treg cell homeostasis. Science.

[52] Brown DG, Rao S, Weir TL, O’Malia J, Bazan M, Brown RJ, Ryan EP (2016). Metabolomics and metabolic pathway networks from human colorectal cancers, adjacent mucosa, and stool. Cancer metabolism.

[53] Furusawa Y, Obata Y, Fukuda S, Endo TA, Nakato G, Takahashi D, Nakanishi Y, Uetake C, Kato K, Kato T (2013). Commensal microbe-derived butyrate induces the differentiation of colonic regulatory T cells. Nature.

[54] Arpaia N, Campbell C, Fan X, Dikiy S, van der Veeken J, Deroos P, Liu H, Cross JR, Pfeffer K, Coffer PJ (2013). Metabolites produced by commensal bacteria promote peripheral regulatory T-cell generation. Nature.

[55] Fukata M, Chen A, Vamadevan AS, Cohen J, Breglio K, Krishnareddy S, Hsu D, Xu R, Harpaz N, Dannenberg AJ (2007). Toll-like receptor-4 promotes the development of colitis-associated colorectal tumors. Gastroenterology.

[56] Tye H, Kennedy CL, Najdovska M, McLeod L, McCormack W, Hughes N, Dev A, Sievert W, Ooi CH (2012). Ishikawa T-o: **STAT3-driven upregulation of TLR2 promotes gastric tumorigenesis independent of tumor inflammation**. Cancer cell.

[57] Wu S, Morin PJ, Maouyo D, Sears CL (2003). *Bacteroides fragilis***enterotoxin induces c-Myc expression and cellular proliferation**. Gastroenterology.

[58] Wu S, Rhee K-J, Zhang M, Franco A, Sears CL (2007). *Bacteroides fragilis***toxin stimulates intestinal epithelial cell shedding and γ-secretase-dependent E-cadherin cleavage**. Journal of cell science.

[59] Wu S, Powell J, Mathioudakis N, Kane S, Fernandez E, Sears CL (2004). *Bacteroides fragilis***enterotoxin induces intestinal epithelial cell secretion of interleukin-8 through mitogen-activated protein kinases and a tyrosine kinase-regulated nuclear factor-κB pathway**. Infect Immun.

[60] Arthur JC, Perez-Chanona E, Mühlbauer M, Tomkovich S, Uronis JM, Fan T-J, Campbell BJ, Abujamel T, Dogan B, Rogers AB (2012). Intestinal inflammation targets cancer-inducing activity of the microbiota. science.

[61] Yamtich J, Nemec AA, Keh A, Sweasy JB (2012). A germline polymorphism of DNA polymerase beta induces genomic instability and cellular transformation. PLoS Genet.

[62] Wang X, Huycke MM (2007). **Extracellular superoxide production by***Enterococcus faecalis***promotes chromosomal instability in mammalian cells**. Gastroenterology.

[63] Nougayrède J-P, Homburg S, Taieb F, Boury M, Brzuszkiewicz E, Gottschalk G, Buchrieser C, Hacker J, Dobrindt U, Oswald E (2006). Escherichia coli induces DNA double-strand breaks in eukaryotic cells. Science.

[64] Nešić D, Hsu Y, Stebbins CE (2004). Assembly and function of a bacterial genotoxin. Nature.

[65] Ge Z, Rogers AB, Feng Y, Lee A, Xu S, Taylor NS, Fox JG (2007). Bacterial cytolethal distending toxin promotes the development of dysplasia in a model of microbially induced hepatocarcinogenesis. Cellular microbiology.

[66] Guidi R, Guerra L, Levi L, Stenerlöw B, Fox JG, Josenhans C, Masucci MG, Frisan T (2013). Chronic exposure to the cytolethal distending toxins of G ram-negative bacteria promotes genomic instability and altered DNA damage response. Cellular microbiology.

[67] Graillot V, Dormoy I, Dupuy J, Shay JW, Huc L, Mirey G, Vignard J (2016). Genotoxicity of cytolethal distending toxin (CDT) on isogenic human colorectal cell lines: potential promoting effects for colorectal carcinogenesis. Front Cell Infect Microbiol.

[68] Ray D, Kidane D (2016). Gut microbiota imbalance and base excision repair dynamics in colon cancer. J Cancer.

[69] Brevik A, Joshi AD, Corral R, Onland-Moret NC, Siegmund KD, Le Marchand L, Baron JA, Martinez ME, Haile RW, Ahnen DJ (2010). Polymorphisms in base excision repair genes as colorectal cancer risk factors and modifiers of the effect of diets high in red meat. Cancer Epidemiology Prevention Biomarkers.

[70] Kraft C, Suerbaum S (2005). **Mutation and recombination in***Helicobacter pylori*: **mechanisms and role in generating strain diversity**. International journal of medical microbiology.

[71] Yadegar A, Mohabati Mobarez A, Zali MR (2019). **Genetic diversity and amino acid sequence polymorphism in***Helicobacter pylori***CagL hypervariable motif and its association with virulence markers and gastroduodenal diseases**. Cancer medicine.

[72] Doorakkers E, Lagergren J, Engstrand L, Brusselaers N. **Eradication of** *Helicobacter pylori***and gastric cancer: a systematic review and meta-analysis of cohort studies**. *JNCI: Journal of the National Cancer Institute* 2016, 108(9). 10.1093/jnci/djw132 27416750

[73] de Melo FF, Rocha GA, Rocha AMC, Teixeira KN, Pedroso SHSP, Junior JBP, de Castro LPF, Cabral MMDÁ, Carvalho SD, Bittencourt PFS. **Th1 immune response to** *H*. *pylori***infection varies according to the age of the patients and influences the gastric inflammatory patterns**. Int J Med Microbiol. 2014;304(3–4):300–6. 10.1016/j.ijmm.2013.11.001 24373859

[74] Uno K, Kato K, Atsumi T, Suzuki T, Yoshitake J, Morita H, Ohara S, Kotake Y, Shimosegawa T, Yoshimura T (2007). **Toll-like receptor (TLR) 2 induced through TLR4 signaling initiated by***Helicobacter pylori***cooperatively amplifies iNOS induction in gastric epithelial cells**. American Journal of Physiology-Gastrointestinal Liver Physiology.

[75] Satoh T, Akira S. Toll-like receptor signaling and its inducible proteins. Microbiology spectrum. 2016;4(6):4.6. 41. 10.1128/microbiolspec.MCHD-0040-2016 28084212

[76] Nejati S, Karkhah A, Darvish H, Validi M, Ebrahimpour S, Nouri HR (2018). **Influence of***Helicobacter pylori***virulence factors CagA and VacA on pathogenesis of gastrointestinal disorders**. Microb Pathog.

[77] Kawasaki T, Kawai T (2014). Toll-like receptor signaling pathways. Frontiers in immunology.

[78] Devi S, Rajakumara E, Ahmed N (2015). **Induction of Mincle by***Helicobacter pylori***and consequent anti-inflammatory signaling denote a bacterial survival strategy**. Scientific reports.

[79] Karkhah A, Ebrahimpour S, Rostamtabar M, Koppolu V, Darvish S, Vasigala VKR, Validi M, Nouri HR (2019). *Helicobacter pylori***evasion strategies of the host innate and adaptive immune responses to survive and develop gastrointestinal diseases**. Microbiological research.

[80] Huang XM, Liu XS, Lin XK, Yu H, Sun JY, Liu XK, Chen C, Jin HL, Zhang GE, Shi XX (2014). Role of plasmacytoid dendritic cells and inducible costimulator-positive regulatory T cells in the immunosuppression microenvironment of gastric cancer. Cancer Sci.

[81] Moyat M, Velin D (2014). **Immune responses to***Helicobacter pylori***infection**. World journal of gastroenterology: WJG.

[82] Udhayakumar G, Jayanthi V, Devaraj N, Devaraj H. **Interaction of MUC1 with β-catenin modulates the Wnt target Gene cyclinD1 in** *H*. *pylori***‐induced gastric cancer**. Molecular Carcinogenesis: Published in cooperation with the University of Texas MD Anderson Cancer Center. 2007;46(9):807–17. 10.1002/mc.20311 17393422

[83] Sato F, Meltzer SJ (2006). CpG island hypermethylation in progression of esophageal and gastric cancer. Cancer: Interdisciplinary International Journal of the American Cancer Society.

[84] Sitaraman R (2014). *Helicobacter pylori***DNA methyltransferases and the epigenetic field effect in cancerization**. Frontiers in microbiology.

[85] Bakhti SZ, Latifi-Navid S, Zahri S, Bakhti FS, Hajavi N, Yazdanbod A (2018). **Are***Helicobacter pylori***highly cytotoxic genotypes and cardia gastric adenocarcinoma linked? Lessons from Iran**. Cancer Biomarkers.

[86] Abdi E, Latifi-Navid S, Zahri S, Yazdanbod A, Safaralizadeh R (2017). *Helicobacter pylori***genotypes determine risk of non-cardia gastric cancer and intestinal- or diffuse-type GC in Ardabil: A very high-risk area in Northwestern Iran**. Microb Pathog.

[87] Bakhti SZ, Latifi-Navid S, Mohammadi S, Zahri S, Bakhti FS, Feizi F, Yazdanbod A, Siavoshi F (2016). **Relevance of***Helicobacter pylori***vacA 3ʹ‐end Region Polymorphism to Gastric Cancer**. Helicobacter.

[88] Suzuki M, Mimuro H, Kiga K, Fukumatsu M, Ishijima N, Morikawa H, Nagai S, Koyasu S, Gilman RH, Kersulyte D (2009). *Helicobacter pylori***CagA phosphorylation-independent function in epithelial proliferation and inflammation**. Cell Host Microbe.

[89] Murata-Kamiya N, Kurashima Y, Teishikata Y, Yamahashi Y, Saito Y, Higashi H, Aburatani H, Akiyama T, Peek R, Azuma T (2007). *Helicobacter pylori***CagA interacts with E-cadherin and deregulates the β-catenin signal that promotes intestinal transdifferentiation in gastric epithelial cells**. Oncogene.

[90] Brandt S, Kwok T, Hartig R, König W, Backert S: **NF**-κ**B activation and potentiation of proinflammatory responses by the** Helicobacter pylori **CagA protein**. *Proceedings of the National Academy of Sciences* 2005, **102**(26):9300–9305. 10.1073/pnas.0409873102 PMC1166591 15972330

[91] Wei J, Noto JM, Zaika E, Romero-Gallo J, Piazuelo MB, Schneider B, El-Rifai W, Correa P, Peek RM, Zaika AI (2015). Bacterial CagA protein induces degradation of p53 protein in a p14ARF-dependent manner. Gut.

[92] Bessede E, Staedel C, Amador LA, Nguyen P, Chambonnier L, Hatakeyama M, Belleannee G, Megraud F, Varon C (2014). *Helicobacter pylori***generates cells with cancer stem cell properties via epithelial–mesenchymal transition-like changes**. Oncogene.

[93] Kountouras J, Kapetanakis N, Zavos C, Polyzos S, Romiopoulos I, Tsiaousi E, Anastasiadou K, Giorgakis N, Vardaka E, Nikolaidou C (2015). *Helicobacter pylori***might contribute to cancer and/or bone marrow-derived stem cell-related gastrointestinal oncogenesis**. Oncogene.

[94] Bessede E, Dubus P, Megraud F, Varon C (2015). *Helicobacter pylori***infection and stem cells at the origin of gastric cancer**. Oncogene.

[95] Bartfeld S, Bayram T, van de Wetering M, Huch M, Begthel H, Kujala P, Vries R, Peters PJ, Clevers H (2015). In vitro expansion of human gastric epithelial stem cells and their responses to bacterial infection. Gastroenterology.

[96] Cristescu R, Lee J, Nebozhyn M, Kim K-M, Ting JC, Wong SS, Liu J, Yue YG, Wang J, Yu K (2015). Molecular analysis of gastric cancer identifies subtypes associated with distinct clinical outcomes. Nature medicine.

[97] Yang F, Xu Y, Liu C, Ma C, Zou S, Xu X, Jia J, Liu Z (2018). **NF-κB/miR-223-3p/ARID1A axis is involved in***Helicobacter pylori***CagA-induced gastric carcinogenesis and progression**. Cell death disease.

[98] Boncristiano M, Paccani SR, Barone S, Ulivieri C, Patrussi L, Ilver D, Amedei A, D’Elios MM, Telford JL, Baldari CT (2003). **The***Helicobacter pylori***vacuolating toxin inhibits T cell activation by two independent mechanisms**. The Journal of experimental medicine.

[99] Sundrud MS, Torres VJ, Unutmaz D, Cover TL: **Inhibition of primary human T cell proliferation by** Helicobacter pylori **vacuolating toxin** (**VacA**) **is independent of VacA effects on IL**-**2 secretion**. *Proceedings of the National Academy of Sciences* 2004, **101**(20):7727–7732. 10.1073/pnas.0401528101 PMC419674 15128946

[100] Gebert B, Fischer W, Weiss E, Hoffmann R, Haas R (2003). *Helicobacter pylori***vacuolating cytotoxin inhibits T lymphocyte activation**. Science.

[101] Oertli M, Noben M, Engler DB, Semper RP, Reuter S, Maxeiner J, Gerhard M, Taube C, Müller A: Helicobacter pylori γ-**glutamyl transpeptidase and vacuolating cytotoxin promote gastric persistence and immune tolerance**. *Proceedings of the National Academy of Sciences* 2013, **110**(8):3047–3052. 10.1073/pnas.1211248110 PMC3581963 23382221

[102] Yang J, Zhou X, Liu X, Ling Z, Ji F. **Role of the gastric microbiome in gastric cancer: from carcinogenesis to treatment**. *Frontiers in Microbiology* 2021, 12. 10.3389/fmicb.2021.641322 PMC8005548 33790881

[103] Hotchin NA, Cover TL, Akhtar N (2000). Cell Vacuolation Induced by the VacA Cytotoxin ofHelicobacter pylori Is Regulated by the Rac1 GTPase. J Biol Chem.

[104] Suzuki J, Ohnsihi H, Shibata H, Wada A, Hirayama T, Iiri T, Ueda N, Kanamaru C, Tsuchida T, Mashima H (2001). **Dynamin is involved in human epithelial cell vacuolation caused by the***Helicobacter pylori***–produced cytotoxin VacA**. J Clin Investig.

[105] Mashima H, Suzuki J, Hirayama T, Yoshikumi Y, Ohno H, Ohnishi H, Yasuda H, Fujita T, Omata M (2008). **Involvement of vesicle-associated membrane protein 7 in human gastric epithelial cell vacuolation induced by***Helicobacter pylori***-produced VacA**. Infect Immun.

[106] Yahiro K, Akazawa Y, Nakano M, Suzuki H, Hisatune J, Isomoto H, Sap J, Noda M, Moss J, Hirayama T (2015). *Helicobacter pylori***VacA induces apoptosis by accumulation of connexin 43 in autophagic vesicles via a Rac1/ERK-dependent pathway**. Cell death discovery.

[107] Ricci V (2016). **Relationship between VacA toxin and host cell autophagy in***Helicobacter pylori***infection of the human stomach: a few answers, many questions**. Toxins.

[108] Galmiche A, Rassow J (2010). **Targeting of***Helicobacter pylori***VacA to mitochondria**. Gut microbes.

[109] Willhite DC, Blanke SR (2004). *Helicobacter pylori***vacuolating cytotoxin enters cells, localizes to the mitochondria, and induces mitochondrial membrane permeability changes correlated to toxin channel activity**. Cellular microbiology.

[110] Jain P, Luo Z-Q, Blanke SR: Helicobacter pylori **vacuolating cytotoxin A** (**VacA**) **engages the mitochondrial fission machinery to induce host cell death**. *Proceedings of the National Academy of Sciences* 2011, **108**(38):16032–16037. 10.1073/pnas.1105175108 PMC3179038 21903925

[111] Caputo R, Tuccillo C, Manzo BA, Zarrilli R, Tortora G, Blanco CDV, Ricci V, Ciardiello F, Romano M (2003). *Helicobacter pylori***VacA toxin up-regulates vascular endothelial growth factor expression in MKN 28 gastric cells through an epidermal growth factor receptor-, cyclooxygenase-2-dependent mechanism**. Clinical cancer research.

[112] Liu N, Zhou N, Chai N, Liu X, Jiang H, Wu Q, Li Q (2016). *Helicobacter pylori***promotes angiogenesis depending on Wnt/beta-catenin-mediated vascular endothelial growth factor via the cyclooxygenase-2 pathway in gastric cancer**. BMC Cancer.

[113] Ki M-R, Lee H-R, Goo M-J, Hong I-H, Do S-H, Jeong D-H, Yang H-J, Yuan D-W, Park J-K, Jeong K-S (2008). Differential regulation of ERK1/2 and p38 MAP kinases in VacA-induced apoptosis of gastric epithelial cells. American Journal of Physiology-Gastrointestinal Liver Physiology.

[114] Song X, Xin N, Wang W, Zhao C. **Wnt/β-catenin, an oncogenic pathway targeted by** *H*. *pylori***in gastric carcinogenesis**. Oncotarget. 2015;6(34):35579. 10.18632/oncotarget.5758 PMC4742126 26417932

[115] Nakayama M, Hisatsune J, Yamasaki E, Isomoto H, Kurazono H, Hatakeyama M, Azuma T, Yamaoka Y, Yahiro K, Moss J (2009). *Helicobacter pylori***VacA-induced inhibition of GSK3 through the PI3K/Akt signaling pathway**. Journal of biological chemistry.

[116] Beigier-Bompadre M, Moos V, Belogolova E, Allers K, Schneider T, Churin Y, Ignatius R, Meyer TF, Aebischer T (2011). **Modulation of the CD4 + T-cell response by***Helicobacter pylori***depends on known virulence factors and bacterial cholesterol and cholesterol α-glucoside content**. J Infect Dis.

[117] Pereira-Marques J, Ferreira RM, Pinto-Ribeiro I, Figueiredo C. *Helicobacter pylori***infection, the gastric microbiome and gastric cancer**. *Helicobacter pylori in Human Diseases* 2019:195–210. 10.1007/5584_2019_366 31016631

[118] Shimizu T, Chiba T, Marusawa H. *Helicobacter pylori***-mediated genetic instability and gastric carcinogenesis**. *Molecular Pathogenesis and Signal Transduction by Helicobacter pylori* 2017:305–323. 10.1007/978-3-319-50520-6_13 28124159

[119] Gobert AP, Wilson KT (2017). **Polyamine-and NADPH-dependent generation of ROS during***Helicobacter pylori***infection: a blessing in disguise**. Free Radic Biol Med.

[120] Chochi K, Ichikura T, Kinoshita M, Majima T, Shinomiya N, Tsujimoto H, Kawabata T, Sugasawa H, Ono S, Seki S (2008). *Helicobacter pylori***augments growth of gastric cancers via the lipopolysaccharide-toll-like receptor 4 pathway whereas its lipopolysaccharide attenuates antitumor activities of human mononuclear cells**. Clin Cancer Res.

[121] Kidd M, Tang L, Schmid S, Lauffer J, Louw J, Modlin I (2000). *Helicobacter pylori***lipopolysaccharide alters ECL cell DNA synthesis via a CD14 receptor and polyamine pathway in mastomys**. Digestion.

[122] Schulz C, Schütte K, Koch N, Vilchez-Vargas R, Wos-Oxley ML, Oxley AP, Vital M, Malfertheiner P, Pieper DH (2018). The active bacterial assemblages of the upper GI tract in individuals with and without Helicobacter infection. Gut.

[123] Bakhti SZ, Latifi-Navid S (2021). **Oral microbiota and***Helicobacter pylori***in gastric carcinogenesis: what do we know and where next?**. BMC microbiology.

[124] Sheh A, Fox JG (2013). **The role of the gastrointestinal microbiome in***Helicobacter pylori***pathogenesis**. Gut microbes.

[125] Wang D, Li Y, Zhong H, Ding Q, Lin Y, Tang S, Zong Y, Wang Q, Zhang X, Yang H (2019). **Alterations in the human gut microbiome associated with***Helicobacter pylori***infection**. FEBS open bio.

[126] Bühling A, Radun D, Müller W, Malfertheiner P (2001). Influence of anti-Helicobacter triple‐therapy with metronidazole, omeprazole and clarithromycin on intestinal microflora. Aliment Pharmacol Ther.

[127] Myllyluoma E, Ahlroos T, Veijola L, Rautelin H, Tynkkynen S, Korpela R (2007). **Effects of anti-***Helicobacter pylori***treatment and probiotic supplementation on intestinal microbiota**. Int J Antimicrob Agents.

[128] Heimesaat MM, Fischer A, Plickert R, Wiedemann T, Loddenkemper C, Göbel UB, Bereswill S, Rieder G (2014). *Helicobacter pylori***induced gastric immunopathology is associated with distinct microbiota changes in the large intestines of long-term infected Mongolian gerbils**. PloS one.

[129] Franceschi F, Zuccalà G, Roccarina D, Gasbarrini A (2014). **Clinical effects of***Helicobacter pylori***outside the stomach**. Nature Reviews Gastroenterology Hepatology.

[130] Iino C, Shimoyama T, Chinda D, Arai T, Chiba D, Nakaji S, Fukuda S (2018). **Infection of***Helicobacter pylori***and atrophic gastritis influence Lactobacillus in gut microbiota in a Japanese population**. Frontiers in immunology.

[131] Benavides-Ward A, Vasquez-Achaya F, Silva-Caso W, Aguilar-Luis MA, Mazulis F, Urteaga N, del Valle-Mendoza J: Helicobacter pylori **and its relationship with variations of gut microbiota in asymptomatic children between 6 and 12 years**. *BMC research notes* 2018, **11**(1):468. 10.1186/s13104-018-3565-5 PMC6043948 30005690

[132] Dash NR, Khoder G, Nada AM, Al Bataineh MT (2019). **Exploring the impact of***Helicobacter pylori***on gut microbiome composition**. PloS one.

[133] Li TH, Qin Y, Sham PC, Lau K, Chu K-M, Leung WK. **Alterations in gastric microbiota after** *H*. *pylori***eradication and in different histological stages of gastric carcinogenesis**. Scientific reports. 2017;7(1):1–8. 10.1038/srep44935 PMC5359573 28322295

[134] Schmitz JM, Durham CG, Schoeb TR, Soltau TD, Wolf KJ, Tanner SM, McCracken VJ, Lorenz RG (2011). *Helicobacter felis***–associated gastric disease in microbiota-restricted mice**. Journal of Histochemistry Cytochemistry.

[135] Skoog EC, Lindberg M, Lindén SK (2011). **Strain-Dependent Proliferation in Response to Human Gastric Mucin and Adhesion Properties of***Helicobacter pylori***are not Affected by Co‐isolated Lactobacillus sp**. Helicobacter.

[136] Llorca L, Pérez-Pérez G, Urruzuno P, Martinez MJ, Iizumi T, Gao Z, Sohn J, Chung J, Cox L, Simón-Soro A (2017). **Characterization of the gastric microbiota in a pediatric population according to***Helicobacter pylori***status**. Pediatr Infect Dis J.

[137] Andersson AF, Lindberg M, Jakobsson H, Bäckhed F, Nyrén P, Engstrand L (2008). Comparative analysis of human gut microbiota by barcoded pyrosequencing. PloS one.

[138] Li J, Perez-Perez GI (2018). *Helicobacter pylori***the latent human pathogen or an ancestral commensal organism**. Frontiers in microbiology.

[139] Vinasco K, Mitchell HM, Kaakoush NO, Castano-Rodriguez N (2019). Microbial carcinogenesis: Lactic acid bacteria in gastric cancer. Biochimica et Biophysica Acta (BBA)-Reviews on Cancer.

[140] Bimczok D, Clements R, Waites K, Novak L, Eckhoff D, Mannon P, Smith P, Smythies L. **Human primary gastric dendritic cells induce a Th1 response to** *H*. *pylori*. Mucosal immunology. 2010;3(3):260–9. 10.1038/mi.2010.10 PMC3683837 20237463

[141] Wang L, Xin Y, Zhou J, Tian Z, Liu C, Yu X, Meng X, Jiang W, Zhao S, Dong Q (2020). Gastric mucosa-associated microbial signatures of early gastric cancer. Frontiers in microbiology.

[142] Chen L, Xu W, Lee A, He J, Huang B, Zheng W, Su T, Lai S, Long Y, Chu H (2018). **The impact of***Helicobacter pylori***infection, eradication therapy and probiotic supplementation on gut microenvironment homeostasis: An open-label, randomized clinical trial**. EBioMedicine.

[143] Cadamuro ACT, Rossi AFT, Maniezzo NM, Silva AE (2014). *Helicobacter pylori***infection: host immune response, implications on gene expression and microRNAs**. World Journal of Gastroenterology: WJG.

[144] Tamura A, Fujioka T, Nasu M (2002). **Relation of***Helicobacter pylori***infection to plasma vitamin B12, folic acid, and homocysteine levels in patients who underwent diagnostic coronary arteriography**. Am J Gastroenterol.

[145] Shuval-Sudai O, Granot E (2003). **An association between***Helicobacter pylori***infection and serum vitamin B12 levels in healthy adults**. Journal of clinical gastroenterology.

[146] LeBlanc JG, Milani C, De Giori GS, Sesma F, Van Sinderen D, Ventura M (2013). Bacteria as vitamin suppliers to their host: a gut microbiota perspective. Curr Opin Biotechnol.

[147] Stopeck A (2000). **Links between***Helicobacter pylori***infection, cobalamin deficiency, and pernicious anemia**. Arch Intern Med.

[148] Miranti EH, Stolzenberg-Solomon R, Weinstein SJ, Selhub J, Männistö S, Taylor PR, Freedman ND, Albanes D, Abnet CC, Murphy G (2017). Low vitamin B12 increases risk of gastric cancer: A prospective study of one‐carbon metabolism nutrients and risk of upper gastrointestinal tract cancer. International journal of cancer.

[149] Carmel R, Aurangzeb I, Qian D (2001). **Associations of food-cobalamin malabsorption with ethnic origin, age**, *Helicobacter pylori***infection, and serum markers of gastritis**. Am J Gastroenterol.

[150] Kaptan K, Beyan C, Ural AU, Cetin T, Avcu F, Gülşen M, Finci R, Yalçín A: Helicobacter pylori**—is it a novel causative agent in vitamin B12 deficiency?** *Archives of Internal Medicine* 2000, **160**(9):1349–1353. 10.1001/archinte.160.9.1349 10809040

[151] Andrès E, Loukili NH, Noel E, Kaltenbach G, Abdelgheni MB, Perrin AE, Noblet-Dick M, Maloisel F, Schlienger J-L, Blicklé J-F (2004). Vitamin B12 (cobalamin) deficiency in elderly patients. Cmaj.

[152] Bi H, Zhu L, Jia J, Zeng L, Cronan JE (2016). **Unsaturated fatty acid synthesis in the gastric pathogen***Helicobacter pylori***proceeds via a backtracking mechanism**. Cell chemical biology.

[153] Ktsoyan ZA, Beloborodova NV, Sedrakyan AM, Osipov GA, Khachatryan ZA, Kelly D, Manukyan GP, Arakelova KA, Hovhannisyan AI, Arakelyan AA (2011). Profiles of microbial fatty acids in the human metabolome are disease-specific. Front Microbiol.

[154] Rooks MG, Garrett WS (2016). Gut microbiota, metabolites and host immunity. Nature reviews immunology.

[155] Igarashi K, Kashiwagi K (2010). Modulation of cellular function by polyamines. Int J Biochem Cell Biol.

[156] Soda K (2011). The mechanisms by which polyamines accelerate tumor spread. Journal of Experimental Clinical Cancer Research.

[157] Yang J, Yu J (2018). The association of diet, gut microbiota and colorectal cancer: what we eat may imply what we get. Protein cell.

[158] Singh N, Gurav A, Sivaprakasam S, Brady E, Padia R, Shi H, Thangaraju M, Prasad PD, Manicassamy S, Munn DH (2014). Activation of Gpr109a, receptor for niacin and the commensal metabolite butyrate, suppresses colonic inflammation and carcinogenesis. Immunity.

[159] Thangaraju M, Cresci GA, Liu K, Ananth S, Gnanaprakasam JP, Browning DD, Mellinger JD, Smith SB, Digby GJ, Lambert NA (2009). GPR109A is a G-protein–coupled receptor for the bacterial fermentation product butyrate and functions as a tumor suppressor in colon. Cancer research.

[160] Donohoe DR, Collins LB, Wali A, Bigler R, Sun W, Bultman SJ (2012). The Warburg effect dictates the mechanism of butyrate-mediated histone acetylation and cell proliferation. Molecular cell.

[161] Donohoe DR, Holley D, Collins LB, Montgomery SA, Whitmore AC, Hillhouse A, Curry KP, Renner SW, Greenwalt A, Ryan EP (2014). A gnotobiotic mouse model demonstrates that dietary fiber protects against colorectal tumorigenesis in a microbiota-and butyrate-dependent manner. Cancer discovery.

[162] Clarke JM, Topping DL, Bird AR, Young GP, Cobiac L (2008). Effects of high-amylose maize starch and butyrylated high-amylose maize starch on azoxymethane-induced intestinal cancer in rats. Carcinogenesis.

[163] Belcheva A, Irrazabal T, Robertson SJ, Streutker C, Maughan H, Rubino S, Moriyama EH, Copeland JK, Surendra A, Kumar S (2014). Gut microbial metabolism drives transformation of MSH2-deficient colon epithelial cells. Cell.

[164] Zhang S, Shi D, Li M, Li Y, Wang X, Li W (2019). The relationship between gastric microbiota and gastric disease. Scand J Gastroenterol.

[165] Yu G, Torres J, Hu N, Medrano-Guzman R, Herrera-Goepfert R, Humphrys MS, Wang L, Wang C, Ding T, Ravel J (2017). Molecular characterization of the human stomach microbiota in gastric cancer patients. Front Cell Infect Microbiol.

[166] Jones M, Mercante RW, Neish JS. A: Reactive oxygen production induced by the gut microbiota: pharmacotherapeutic implications. Curr Med Chem. 2012;19(10):1519–29. 10.2174/092986712799828283 PMC4269156 22360484

[167] Faubert B, Li KY, Cai L, Hensley CT, Kim J, Zacharias LG, Yang C, Do QN, Doucette S, Burguete D (2017). Lactate metabolism in human lung tumors. Cell.

[168] San-Millán I, Brooks GA (2017). Reexamining cancer metabolism: lactate production for carcinogenesis could be the purpose and explanation of the Warburg Effect. Carcinogenesis.

[169] Yeung KT, Yang J (2017). Epithelial–mesenchymal transition in tumor metastasis. Molecular oncology.

[170] Oh B, Kim BS, Kim JW, Kim JS, Koh SJ, Kim BG, Lee KL, Chun J (2016). **The effect of probiotics on gut microbiota during the***Helicobacter pylori***eradication: randomized controlled trial**. Helicobacter.

[171] Fang H-R, Zhang G-Q, Cheng J-Y, Li Z-Y (2019). **Efficacy of Lactobacillus-supplemented triple therapy for***Helicobacter pylori***infection in children: a meta-analysis of randomized controlled trials**. Eur J Pediatrics.

[172] Yu M, Zhang R, Ni P, Chen S, Duan G. **Efficacy of Lactobacillus-supplemented triple therapy for** *H*. *pylori***eradication: A meta-analysis of randomized controlled trials**. PloS one. 2019;14(10):e0223309. 10.1371/journal.pone.0223309 PMC6774518 31577828

[173] Yang YJ, Sheu BS (2012). **Probiotics-containing yogurts suppress***Helicobacter pylori***load and modify immune response and intestinal microbiota in the***Helicobacter pylori***‐infected children**. Helicobacter.

[174] Sakarya S, Gunay N (2014). *Saccharomyces boulardii***expresses neuraminidase activity selective for α2, 3-linked sialic acid that decreases***Helicobacter pylori***adhesion to host cells**. Apmis.

[175] Song H, Zhou L, Liu D, Ge L, Li Y (2019). **Probiotic effect on***Helicobacter pylori***attachment and inhibition of inflammation in human gastric epithelial cells**. Experimental therapeutic medicine.

[176] Wang Z-H, Gao Q-Y, Fang J-Y (2013). **Meta-analysis of the efficacy and safety of Lactobacillus-containing and Bifidobacterium-containing probiotic compound preparation in***Helicobacter pylori***eradication therapy**. J Clin Gastroenterol.

[177] Zheng C, Chen T, Wang Y, Gao Y, Kong Y, Liu Z, Deng X (2019). A randomised trial of probiotics to reduce severity of physiological and microbial disorders induced by partial gastrectomy for patients with gastric cancer. journal of cancer.

[178] Yaghoubi A, Khazaei M, Jalili S, Hasanian SM, Avan A, Soleimanpour S, Cho WC (2020). Bacteria as a double-action sword in cancer. Biochimica et Biophysica Acta (BBA)-Reviews on Cancer.

[179] Soleimanpour S, Hasanian SM, Avan A, Yaghoubi A, Khazaei M (2020). Bacteriotherapy in gastrointestinal cancer. Life sciences.

[180] Hu C, Chen X, Huang Y, Chen Y (2018). Synergistic effect of the pro-apoptosis peptide kla-TAT and the cationic anticancer peptide HPRP-A1. Apoptosis.

[181] Mai Xt, Huang J, Tan J, Huang Y, Chen Y (2015). Effects and mechanisms of the secondary structure on the antimicrobial activity and specificity of antimicrobial peptides. J Pept Sci.

[182] Zhao J, Huang Y, Liu D, Chen Y (2015). Two hits are better than one: synergistic anticancer activity of α-helical peptides and doxorubicin/epirubicin. Oncotarget.

[183] Hao W, Hu C, Huang Y, Chen Y (2019). Coadministration of kla peptide with HPRP-A1 to enhance anticancer activity. Plos one.

[184] Zhao J, Hao X, Liu D, Huang Y, Chen Y (2015). In vitro characterization of the rapid cytotoxicity of anticancer peptide HPRP-A2 through membrane destruction and intracellular mechanism against gastric cancer cell lines. PLoS One.

[185] Noto JM, Peek RM (2017). **The gastric microbiome, its interaction with***Helicobacter pylori*, **and its potential role in the progression to stomach cancer**. PLoS pathogens.

